# *mcPGK1*-dependent mitochondrial import of PGK1 promotes metabolic reprogramming and self-renewal of liver TICs

**DOI:** 10.1038/s41467-023-36651-5

**Published:** 2023-02-27

**Authors:** Zhenzhen Chen, Qiankun He, Tiankun Lu, Jiayi Wu, Gaoli Shi, Luyun He, Hong Zong, Benyu Liu, Pingping Zhu

**Affiliations:** 1 School of Life Sciences, Zhengzhou University, 100 Kexue Road, Zhengzhou, Henan 450001 China; 2 School of Medicine, Nankai University, 94 Weijin Road, Tianjin, 300071 China; 3 Department of Pathophysiology, School of Basic Medical Sciences, Zhengzhou University, Zhengzhou, Henan 450001 China; 4 Department of Oncology, The First Affiliated Hospital of Zhengzhou University, NO.1 Eastern Jianshe Road, Zhengzhou, 450052 Henan China; 5 Research Center of Basic Medicine, Academy of Medical Sciences, Zhengzhou University, Zhengzhou, Henan China

**Keywords:** Non-coding RNAs, Cancer stem cells, Cancer metabolism

## Abstract

Liver tumour-initiating cells (TICs) contribute to tumour initiation, metastasis, progression and drug resistance. Metabolic reprogramming is a cancer hallmark and plays vital roles in liver tumorigenesis. However, the role of metabolic reprogramming in TICs remains poorly explored. Here, we identify a mitochondria-encoded circular RNA, termed *mcPGK1* (mitochondrial circRNA for translocating phosphoglycerate kinase 1), which is highly expressed in liver TICs. *mcPGK1* knockdown impairs liver TIC self-renewal, whereas its overexpression drives liver TIC self-renewal. Mechanistically, *mcPGK1* regulates metabolic reprogramming by inhibiting mitochondrial oxidative phosphorylation (OXPHOS) and promoting glycolysis. This alters the intracellular levels of α-ketoglutarate and lactate, which are modulators in Wnt/β-catenin activation and liver TIC self-renewal. In addition, *mcPGK1* promotes PGK1 mitochondrial import via TOM40 interactions, reprogramming metabolism from oxidative phosphorylation to glycolysis through PGK1-PDK1-PDH axis. Our work suggests that mitochondria-encoded circRNAs represent an additional regulatory layer controlling mitochondrial function, metabolic reprogramming and liver TIC self-renewal.

## Abstract (web summary)

Metabolic reprogramming plays vital roles in tumorigenesis. Here, Chen et al. reveal that mitochondria-encoded mcPGK1 drives the mitochondrial translocation of PGK1, promoting liver tumorigenesis and TIC self-renewal by switching energy production from OXPHOS to glycolysis.

## Introduction

Liver cancer is a common tumor type and many liver cancer patients have a very poor prognosis, which is largely due to tumor heterogeneity^1^. Accumulating researches have proved that tumor heterogeneity originates from the hierarchic organization of tumor cells that are derived from a small population of cells, termed as tumor initiating cells (TICs) or cancer stem cells (CSCs)^1^. Several markers of liver TICs (or CSCs) have been identified, such as CD133, CD13 and ZIC2^2–4^. Unlike differentiated cancer cells, TICs are resistant to traditional radiotherapy and chemotherapy, and increasing studies demonstrate that TICs are also insensitive to CAR-T and immune checkpoint therapies^5,6^. Liver TICs is regulated by Wnt/β-catenin^7,8^, Notch^9^, Hedgehog^10^ and Hippo/Yap signaling pathways^11^, and these pathways are further accurately modulated. However, the molecular mechanisms of liver TIC function remain elusive.

Circular RNAs (circRNAs) are newly identified regulatory RNA molecules that have emerged as critical modulators in multiple biological processes^12^. Several circRNAs are identified as microRNA sponges, such as ciRS-7/CDR1as^13,14^. Fusion circRNAs and rtcircRNA are involved in tumorigenesis and drug-resistance^15,16^. We have identified *circPan3* and *circKcnt2* as regulators in intestinal stem cell (ISC) and colitis, respectively^17,18^. Moreover, we also revealed some circRNAs involved in the self-renewal regulation of tumor cells and TICs, including *cia-MAF*^19^, *cis-HOX*^20^ and *circREEP3*^21^. Recently, mitochondria-encoded circRNAs have been identified, which are involved in communication between mitochondria and the nucleus^22^. Nonalcoholic steatohepatitis (NASH)-related mitochondrial circRNA *SCAR* (abbreviated for Steatohepatitis-associated circRNA ATP5B Regulator) interacts with ATP5B and inhibits mitochondrial reactive oxygen species (mROS) production and fibroblast activation^23^. However, the functions and regulatory mechanisms of mitochondria-encoded circRNAs in tumorigenesis and TICs are hitherto unclear.

Mitochondria are the key energy factories in almost all eukaryote cells. Mitochondria contain their own DNA, which encodes mitochondria-specific proteins and noncoding RNAs, such as 16 S ribosomal RNA, some transfer RNAs and circRNAs^24^. Mitochondria contain 1000–3000 proteins, most of which are encoded by nuclear DNA and transported from the cytoplasm by mitochondrial translocases, such as the TOM40 complex^25,26^. Many metabolic processes, including oxidative phosphorylation (OXPHOS), fatty acid β-oxidation and the urea cycle, occur in mitochondria^27,28^. These metabolic processes are regulated by various intracellular and extracellular factors. Recently, we have revealed that a mitochondria-located methyltransferase, Mettl4, inhibits mitochondrial transcription, OXPHOS, glycolysis, and mROS production^29^. Morphological and functional alterations of mitochondria are also driven by a variety of external factors, including hypoxia, starvation, infection, and tumorigenesis^30,31^. A hallmark of tumorigenesis is metabolic reprogramming, in which the main metabolic pathway switches from OXPHOS to glycolysis, a process also known as the Warburg effect^32^. One possible necessary of metabolic reprogramming is that some intermediate products of glycolysis provide a material basis for the rapid propagation of tumor cells. Indeed, the activities of glycolysis and OXPHOS are adjusted in cells within various stages of cell cycle. Cells in G1 stage prefer OXPHOS, whereas cells in S stage prefer glycolysis^33^. In addition to energy production, glycolysis and OXPHOS produce various metabolites that regulate multiple intracellular and extracellular biological processes. For example, the production of lactic acid during glycolysis inhibits the activity of T cells, enabling the immune escape of tumor cells^34^. In the present study, we have characterized a mitochondrial circRNA, termed *mcPGK1* (mitochondrial circRNA for translocating phosphor- glycerate kinase 1), which is highly expressed in liver TICs and liver tumors. We found that *mcPGK1* promotes the mitochondrial localization of PGK1, contributes to the metabolic reprogramming from OXPHOS to glycolysis.

## Results

### *McPGK1* is highly expressed in liver TICs

**Fig. 1:**
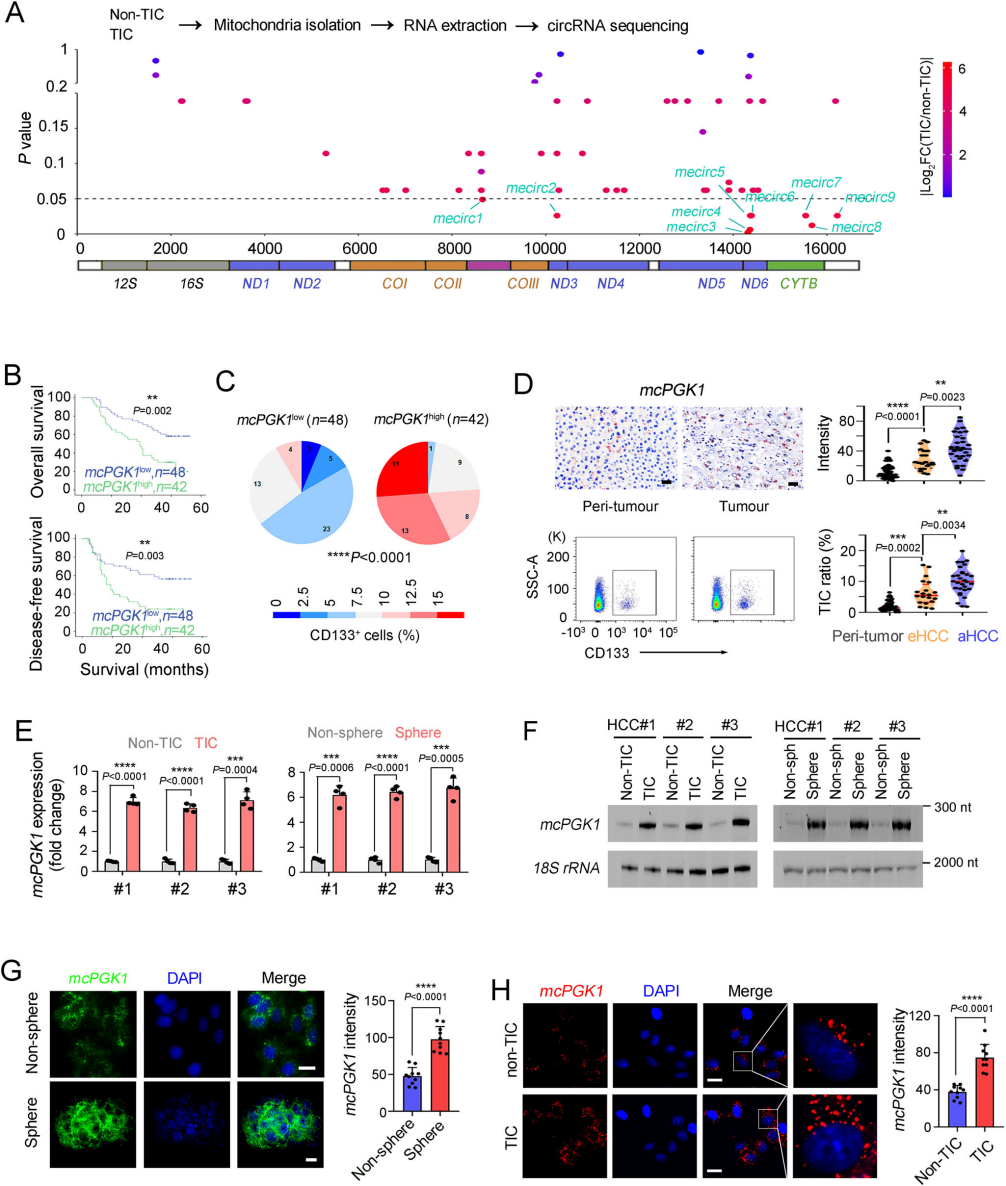
High expression of *mcPGK1* in liver tumor and liver TICs. **A** TICs and non-TICs were used for mitochondria isolation, and mitochondrial encoded circRNAs (mecirc) were identified through circRNA sequencing. Nine mecircRNAs with fold change |FC | > 2 and *P*-value < 0.05 were labeled as mecirc1-9 according to their locus on mitochondrial DNA. **B** Kaplan–Meier survival analysis of *mcPGK1* high-expressing (*mcPGK1*^high^) and *mcPGK1* low-expressing (*mcPGK1*^low^) patients, which were clustered according to the average level of *mcPGK1* intensity in 90 liver tumor tissues (109.6). **C** Percentage distribution of CD133^+^ TICs in *mcPGK1*^low^ (left) and *mcPGK1*^high^ (right) samples. **D** RNAscope detection of 50 peri-tumor, 20 early HCC (eHCC) and 30 advanced HCC (aHCC) samples. Typical RNAscope images, CD133 FACS images and scatter plots were shown. Gating strategy was shown in supplementary Fig. 4E. Scale bars, 30 μm. **E** Quantitative real-time PCR analysis for *mcPGK1* expression in CD133^+^ TICs and spheres. *n* = 4 independent experiments. **F** Northern blot for *mcPGK1* expression in CD133^+^ TICs and CD133^-^ non-TICs (left), or non-spheres and spheres (right). 18 S rRNA is a loading control. **G** Fluorescence in situ hybridization of *mcPGK1* in non-sphere cells and spheres. Typical images were shown in the left panel and *n* = 10 images were taken for statistical analysis of *mcPGK1* intensity (right). Scale bars, 10 μm. **H** RNAscope for *mcPGK1* detection in TICs and non-TICs. Representative images and statistical intensity from *n* = 10 fields were shown. Scale bars, 10 μm. In all panels, data are shown as mean + s.d. **P < 0.01; ***P < 0.001; ****P < 0.0001, by CHISQ Test (**C**) or two-tailed Student’s *T*-test (for **D**, **E**, **G**, **H**). Source data are provided as a Source Data file.

Mitochondrial DNA-encoded circular RNAs (mecircRNAs) emerge as a new type of circRNAs^22^, and have been identified as critical modulators in NASH^23^, but their functions in tumorigenesis and TIC self-renewal are unknown. Here, we sorted CD133^+^ cells from primary liver cancer and proved these cells as liver TICs (Supplementary Fig. 1A–C), and isolated mitochondria from liver TICs and non-TICs for circRNA sequencing. There were 54 mecircRNAs identified in liver cancer cells. Among them, 9 mecircRNAs were differentially expressed (FC > 2, *P*-value < 0.05) in liver TICs and non-TICs, and named mecirc1-9 according to their corresponding locus of mitochondrial DNA (Fig. 1A). Then we designed circRNA-specific primers (Supplementary Fig. 1D) and detected their expression levels in six pairs of liver tumor and peri-tumor samples, and eight mecircRNAs were differently expressed in liver tumors (Supplementary Fig. 1E). Four pairs of TICs and non-TICs were used to further analyze mecircRNA expression, and finally six mecircRNAs (*mecirc4*, *mecirc5*, *mecirc6*, *mecirc7*, *mecirc8*, *mecirc9*) were screened out (Supplementary Fig. 1F, G). RNase R digestion, PCR and DNA-sequencing also confirmed that these six mecircRNAs are circular RNA (Supplementary Fig. 1H, I).

To identify functional mecircRNA, we generated mecircRNA overexpressing cells (Supplementary Fig. 2A, B), followed by TIC detection. *mecirc4*, *mecirc5* and *mecirc8* drove expression of TIC marker CD133, whereas *mecirc5* and *mecirc8* promoted c-Myc expression (Supplementary Fig. 2C). Moreover, sphere formation assay showed that *mecirc5*, *mecirc6* and *mecirc8* were involved in liver TIC self-renewal (Supplementary Fig. 2D), and we focused on *mecirc8* (hereafter termed as *mcPGK1*) for further analysis. *mcPGK1* was generated from *CYTB* locus of mitochondrial DNA (Supplementary Fig. 2E). Then *mcPGK1* specific probe was screened out by RNase H digestion assay and real-time PCR detection (Supplementary Fig. 2F), and confirmed via fluorescence in situ hybridization (Supplementary Fig. 2G). About 400–800 copies of *mcPGK1* were detected in each liver TIC or sphere cell (Supplementary Fig. 3A). Then the subcellular location of *mcPGK1* was measured. *mcPGK1* was enriched in cytosol factions (including mitochondria) but not enriched in nuclear fractions (Supplementary Fig. 3B). Then mitochondria were isolated from cytosol fractions, and *mcPGK1* was predominantly localized in mitochondria and also detectable in cytoplasm (Supplementary Fig. 3C–E). Moreover, total and mitochondrial mcPGK1 were increased in liver tumorigenesis (Supplementary Fig. 3F). We also examined the exact location of *mcPGK1* in mitochondrial fractions isolated with APEX labeling, and found *mcPGK1* was enriched in outer mitochondrial membrane and matrix, and also detectable in intermembrane space (Supplementary Fig. 3G, H). Fraction separation of mitochondria confirmed these results (Supplementary Fig. 3I). These data demonstrated that *mcPGK1* was a mitochondrial DNA-encoded circular RNA and preferentially located in mitochondria.

Tox further evaluate the expression signature of *mcPGK1* in liver tumors, we performed *mcPGK1* in situ hybridization and the results showed that *mcPGK1* was highly expressed in liver tumors (Supplementary Fig. 4A). The expression of *mcPGK1* was correlated with clinical stages, tumor volumes, relapse and survival (Fig. 1B and Supplementary Fig. 4B, C). *mcPGK1* was co-expressed with CD133, a marker of TICs (Supplementary Fig. 4D). Samples with low *mcPGK1* expression harbored fewer TICs, whereas samples with high *mcPGK1* expression harbored more TICs (Fig. 1C). We also validated the upregulation of *mcPGK1* in liver tumors through RNAscope (Fig. 1D). Moreover, *mcPGK1* RNAscope signals were positively related to CD133 ratios, confirming the microarray data (Fig. 1D and Supplementary Fig. 4E, F). The high expression of *mcPGK1* in spheres and CD133^+^ TICs was confirmed by real-time PCR (Fig. 1E), Northern blotting (Fig. 1F), fluorescence in situ hybridization (Fig. 1G and Supplementary Fig. 4H), and RNAscope (Fig. 1H). Cytoplasmic and mitochondrial fractions were also separated from non-TICs, TICs, non-sphere and sphere cells, and *mcPGK1* was proved to be enriched in mitochondrial fractions, especially in mitochondria of sphere cells (Supplementary Fig. 4G). Of note, the mitochondrial levels were comparable between liver TICs and non-TICs (Supplementary Fig. 4I). Taken together, mitochondria-encoded *mcPGK1* is upregulated in liver TICs.

### *McPGK1* drives the self-renewal of liver TIC

**Fig. 2:**
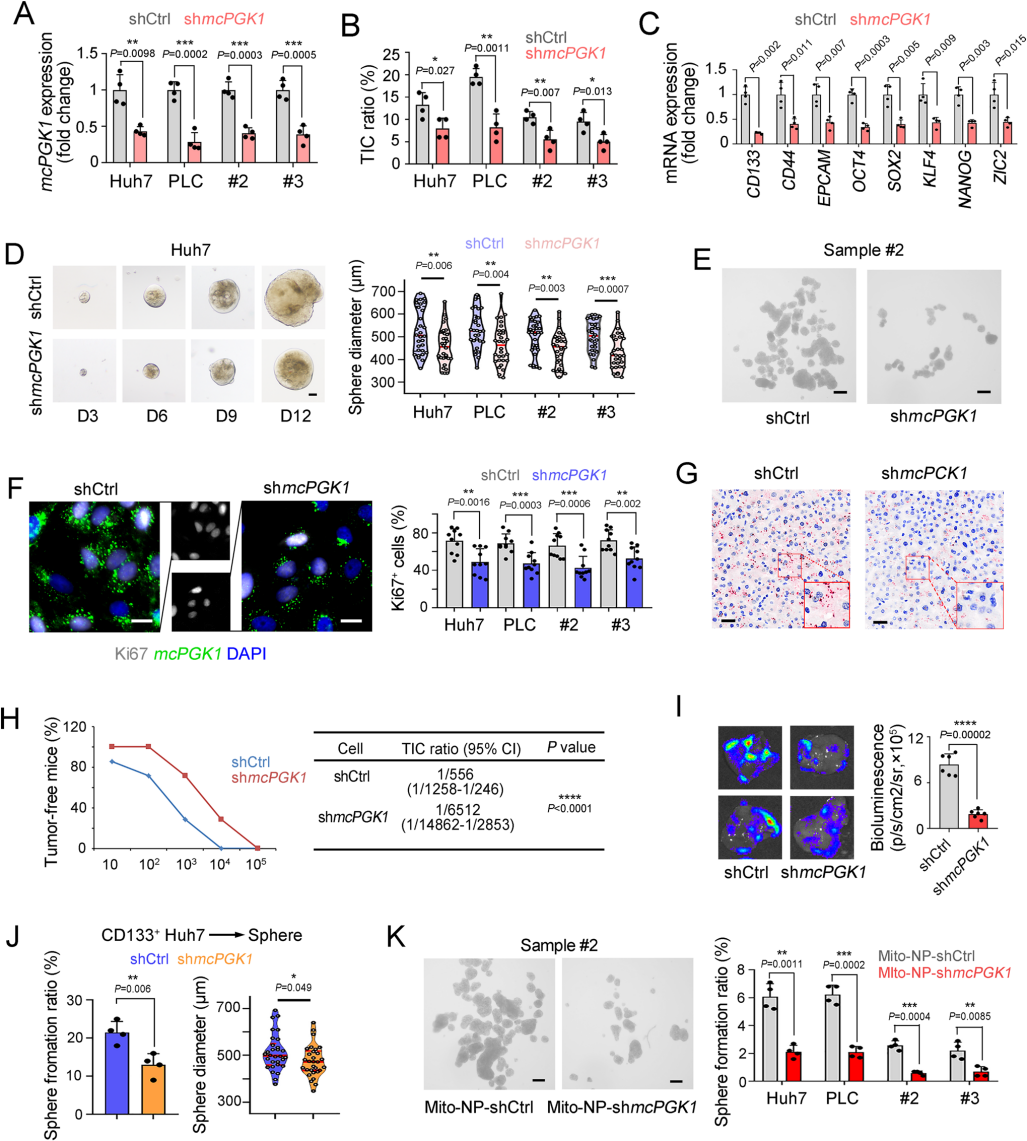
*McPGK1* is required for liver TIC self-renewal. **A** Quantitative real-time PCR to confirm the knockdown efficiency in *mcPGK1* silenced cells. **B** FACS detection of *mcPGK1* silenced cells for CD133 expression. **C** Quantitative real-time PCR analysis of TIC markers and TIC-associated TFs in *mcPGK1* silenced and control cells. **D** Sphere formation of *mcPGK1* silenced and control cells. Typical sphere images were in the left panel and sphere diameters of *n* = 30 spheres from three independent experiments were measured in the right panel. D3, day 3. Scale bars, 100 μm. **E** Sphere formation of *mcPGK1* silenced and control HCC #2 sample cells. Scale bars, 500 μm. **F** *McPGK1* fluorescence in situ hybridization and Ki67 staining in *mcPGK1* silenced and control Huh7 cells. Typical images and Ki67^+^ ratios were shown. For each group, *n* = 10 fields were observed. Scale bars, 10 μm. **G**, **H** Tumor initiation assay of gradient numbers of *mcPGK1* silenced and control cells derived from spheres derived from primary #2 cells. *mcPGK1* detection for knockdown efficiency was shown in G. *n* = 7 6-week-old male BALB/c nude mice were used for H. TIC ratios and *P*-value were calculated by extreme limiting dilution analysis (ELDA) (http://bioinf.wehi.edu.au/software/elda/). Scale bars, 30 μm. **I** 1 × 10^6^ *mcPGK1* knockdown and control luciferase labeled primary #2 cells were used for in vivo propagation. Typical liver images and calculated results were shown. *n* = 6 mice per group. **J** Sphere formation with 1000 CD133^+^ control and sh*mcPGK1* Huh7 cells, which were sorted via FACS assay. **K** Sphere formation with *mcPGK1* knockdown and control cells, for which sh*mcPGK1* and control plasmids were delivered into primary HCC cells derived from sample #2 with mitochondria-targeting nanoparticles (Mito-NP). Scale bars, 500 μm. For **A**, **B**, **C**, **E**, **J**, **K**, *n* = 4 independent experiments. In all panels, data are shown as mean + s.d. *P < 0.05; **P < 0.01; ***P < 0.001; ****P < 0.0001, by two-tailed Student’s *T*-test. Source data are provided as a Source Data file.

We then evaluated the function of *mcPGK1* in liver TICs. First, we detected *mcPGK1* expression levels across several HCC cell lines and primary samples, and found that it was differentially expressed among these cell lines and tissue samples (Supplementary Fig. 5A, B). We then constructed *mcPGK1*-knockdown cells using *mcPGK1* high-expressing cells and sh*mcPGK1* was screened out to specifically target *mcPGK1* but not linear RNA (Fig. 2A and Supplementary Fig. 5C, D). Moreover, *mcPGK1* knockdown decreased both cytoplasmic and mitochondrial *mcPGK1* levels, although knockdown efficiency of mitochondrial *mcPGK1* was lower than cytoplasmic *mcPGK1* (Supplementary Fig. 5E, F). *McPGK1* silenced cells contained fewer liver TICs, and expressed lower levels of TIC markers and TIC-associated genes (Fig. 2B, C). The *mcPGK1* silenced cells also displayed impaired sphere formation and proliferation capacities, but cell apoptosis was not influenced by *mcPGK1* knockdown (Fig. 2D–F, and Supplementary Fig. 5G, H). Moreover, decreased tumor initiation and propagation capacity was detected in *mcPGK1* silenced cells (Fig. 2G–I). We then obtained sh*mcPGK1* CD133^+^ liver TICs and sh*mcPGK1* sphere cells for self-renewal detection, and revealed that *mcPGK1* promotes self-renewal in liver TICs (Fig. 2J and Supplementary Fig. 5I). To further target mitochondrial *mcPGK1* efficiently, we also constructed mitochondria-targeting nanoparticles^25^, and found these nanoparticles can efficiently target mitochondrial *mcPGK1* (Supplementary Fig. 5J). sh*mcPGK1* cells established by mitochondria-targeting nanoparticles also showed impaired sphere formation capacity (Fig. 2K).

We also constructed mcPGK1 overexpressing cells, which also showed mitochondrial location of *mcPGK1* (Supplementary Fig. 6A–C). These cells harbored increased TIC ratios and TIC marker expression (Supplementary Fig. 6D, E). Furthermore, *mcPGK1* overexpression enhanced TIC self-renewal, tumor initiation but not apoptosis (Supplementary Fig. 6F–I). *mcPGK1* was then overexpressed using mitochondria-targeting nanoparticles, and *mcPGK1* overexpression in mitochondria also promoted liver TIC self-renewal (Supplementary Fig. 6J, K). Moreover, *mcPGK1* overexpression also drove tumor propagation in vivo (Supplementary Fig. 6L). Overall, these findings indicate that *mcPGK1* is required for liver TIC self-renewal.

### *McPGK1* reprograms metabolism from OXPHOS to glycolysis

**Fig. 3:**
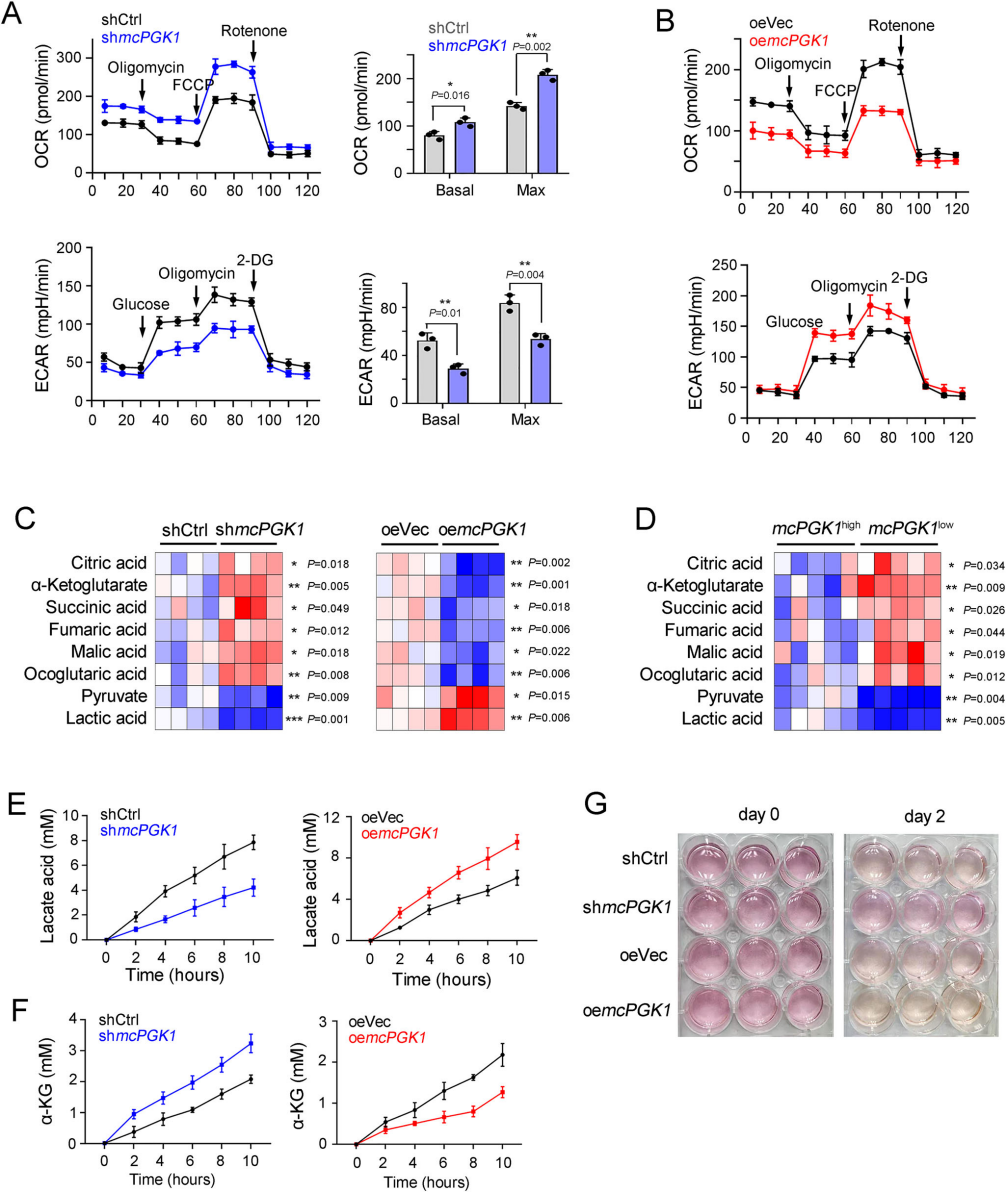
*McPGK1* drives the metabolic reprogramming from OXPHOS to glycolysis. **A**, **B** Oxygen consumption rate (OCR) and extracellular acidification rate (ECAR) of *mcPGK1* silenced (**A**) and overexpressing (**B**) cells. Huh7 cells were used for *mcPGK1* knockdown (**A**) and Hep3B cells were used for *mcPGK1* overexpression (**B**). *n* = 3 independent experiments for each cell. **C** Intracellular levels of the indicated metabolites in *mcPGK1* silenced (left, sample #2) and overexpressing (right, sample #4) panels. *n* = 4 independent experiments. **D** Five *mcPGK1* high-expressing (*mcPGK1*^high^, sample #2, #3, #12, #10, #13) and *mcPGK1* low-expressing (*mcPGK1*^low^, sample #4, #6, #7, #1, #15) samples were used for metabolite detection. **E**, **F** The abundance of lactate (**E**) and α-KG (**F**) in the indicated medium supernatant was measured at the indicated time points. Huh7 and Hep3B were used for *mcPGK1* knockdown and overexpression. *n* = 4 independent experiments. **G** Acidification of the culture medium in *mcPGK1* silenced (Huh7) and overexpressing (Hep3B) cells, as indicated by the color change of the phenol red indicator in the medium to orange/yellow. Typical images were shown representative of *n* = 3 independent experiments. In all panels, data are shown as mean + s.d. *P < 0.05; **P < 0.01; ***P < 0.001, by two-tailed Student’s *T*-test. Source data are provided as a Source Data file.

As the central function of mitochondria is energy metabolism, we evaluated the effects of *mcPGK1* on OXPHOS and glycolysis. OXPHOS activity was enhanced and glycolytic activity was attenuated in *mcPGK1*-silenced cells, whereas the opposite occurred in *mcPGK1*-overexpressing cells, indicating critical roles of *mcPGK1* in metabolic reprogramming shifting from OXPHOS to glycolysis (Fig. 3A, B). Supporting these findings, the OXPHOS metabolite levels were increased and glycolytic metabolite levels were decreased in *mcPGK1*-silenced cells, whereas *mcPGK1*-overexpressing cells contained increased levels of glycolytic metabolites (Fig. 3C). Interestingly, divergent levels of OXPHOS and glycolysis metabolites were detected in primary cells with high or low *mcPGK1* expression (Fig. 3D). These results demonstrate the essential role of *mcPGK1* in metabolic reprogramming of liver TICs.

We then determined the time-course levels of lactic acid and α-KG, two of the main metabolites of glycolysis and OXPHOS, respectively. Lactic acid accumulated rapidly in *mcPGK1*-overexpressing cells, but not in *mcPGK1*-knockdown cells (Fig. 3E). In contrast, α-KG was accumulated rapidly in *mcPGK1*-knockdown cells, but not in *mcPGK1*-overexpressing cells (Fig. 3F). Similarly, the medium of *mcPGK1*-overexpressing cells tended to become orange/yellow, whereas the medium of *mcPGK1*-silenced cells tended to stay red, confirming the role of *mcPGK1* in the acidification of culture medium (Fig. 3G). These data demonstrated that *mcPGK1* was involved in metabolic reprogramming.

### Metabolic reprogramming drives liver TIC function via Wnt pathway

**Fig. 4:**
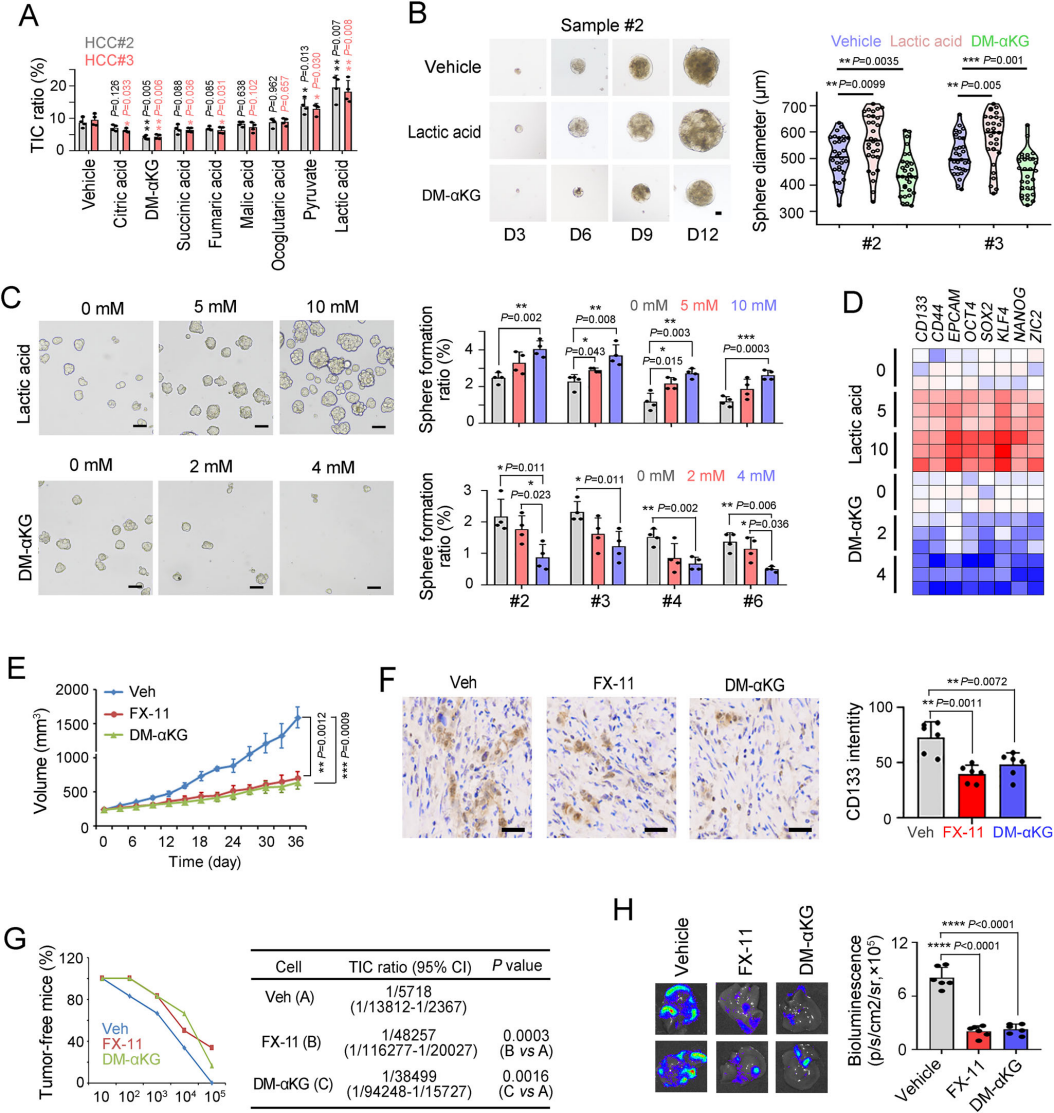
Lactate and α-KG are involved in liver TIC self-renewal. **A** CD133^+^ TIC ratios after 2 days’ treatment with the indicated metabolites were detected via FACS. DM-αKG, cell permeable α-KG. *n* = 4 independent experiments. **B** Typical images and sphere diameters in lactate and DM-αKG treated sample #2 cells. *n* = 30 spheres from three independent experiments were measured. D3, day 3. Scale bars, 100 μm. **C** Sphere formation of sample #2 cells supplemented with indicated levels of lactate and DM-αKG. *n* = 4 independent experiments. Scale bars, 500 μm. **D** Quantitative real-time PCR detection for the indicated gene expression in lactate and DM-αKG treated primary sample #2 cells. *n* = 3 independent experiments. **E** Xenograft tumors were established in BALB/c nude mice and treated with FX-11 or DM-αKG after tumors reached about 250mm^3^, and tumor volume was detected every 3 days. *n* = 6 6-week-old male BALB/c nude mice per group. **F** CD133 immunohistochemistry in Vehicle (Veh), lactic acid production inhibitor FX-11 and DM-αKG treated tumors. *n* = 6 tumors per group, and for each tumor, *n* = 10 images were detected. Scale bars, 50 μm. **G** 10, 1 × 10^2^, 1 × 10^3^, 1 × 10^4^ and 1 × 10^5^ Huh7 sphere cells were injected into BALB/c nude mice for tumor initiation assay. *n* = 6 6-week-old male BALB/c nude mice per group. TIC ratios and *P-*values calculated via ELDA were shown in the right panels. **H** 1 × 10^6^ luciferase labeled primary #2 cells were used for in vivo propagation, and mice were administered with FX-11 (2 mg/kg) or DM-αKG (500 mg/kg). Typical liver images and calculated results were shown. *n* = 6 mice per group. For A, C, *n* = 4 independent experiments. In all panels, data are shown as mean + s.d. *P < 0.05; **P < 0.01; ***P < 0.001; ****P < 0.0001, by two-tailed Student’s *T*-test. Source data are provided as a Source Data file.

The functions of metabolic reprogramming in liver TICs are unknown. Among the metabolites we evaluated, lactic acid and α-KG functioned as stemness modulators in liver TICs (Fig. 4A). Lactic acid drove liver TIC self-renewal, whereas α-KG had an opposite effect (Fig. 4B, C). Lactic acid treatment increased the expression of TIC-associated genes, whereas α-KG elicited opposite effects (Fig. 4D). Then the roles of lactic acid and α-KG in liver tumor propagation and liver TIC self-renewal were examined in vivo. FX-11, an inhibitor of lactic acid production, inhibited liver tumor propagation (Fig. 4E and Supplementary Fig. 7A), decreased the ratios of CD133^+^ liver TICs (Fig. 4F), and impaired the tumor initiation capacity (Fig. 4G), indicating that lactic acid was a driver of liver TIC self-renewal. In contrast with lactic acid, α-KG showed inhibitory effects on liver tumor propagation and liver TIC maintenance (Fig. 4E–G). The modulation of liver tumor propagation by lactic acid and α-KG was confirmed by in vivo luciferase assay (Fig. 4H). These results confirmed that metabolic reprogramming was involved in liver TIC maintenance.

**Fig. 5:**
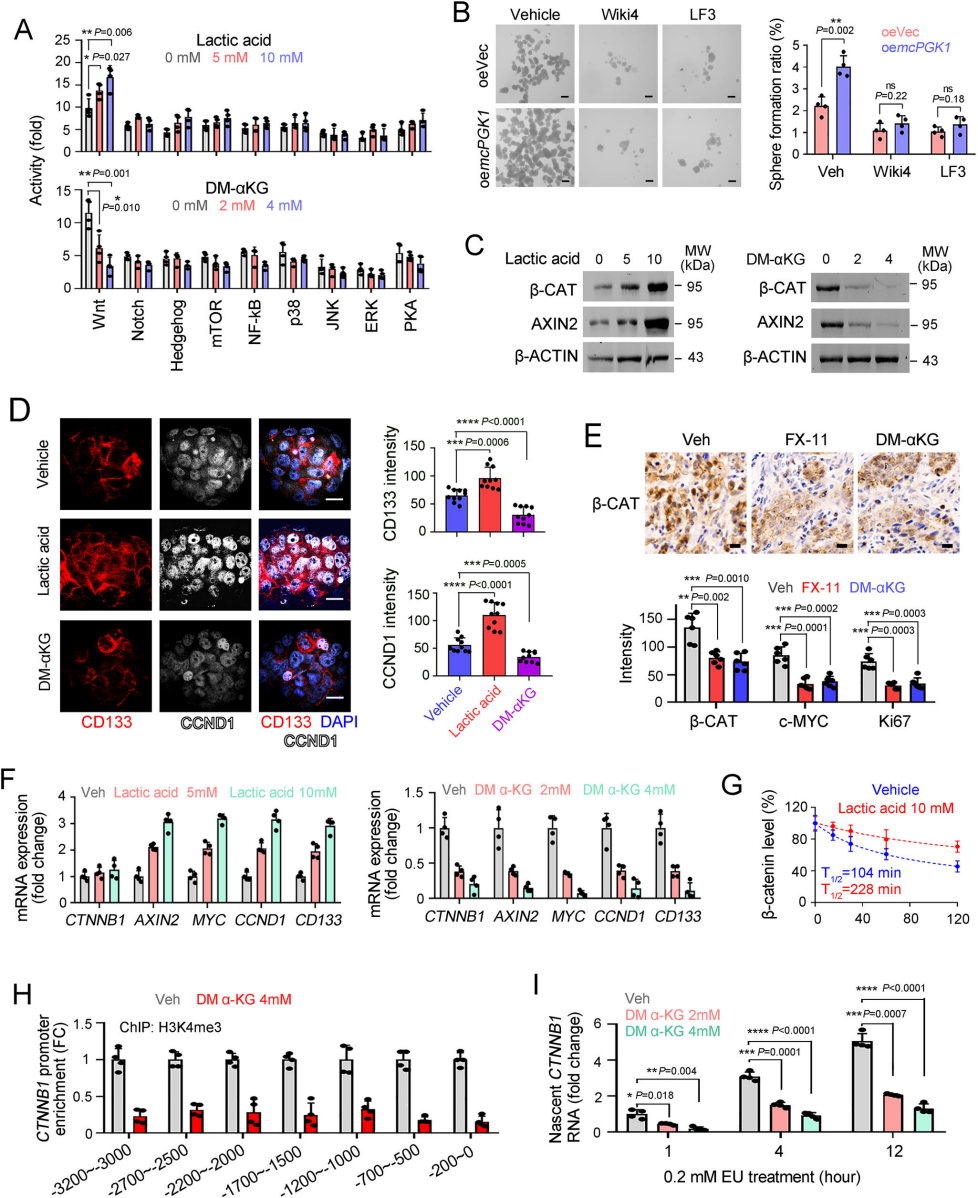
Lactate and α-KG are involved in Wnt/β-catenin activation. **A** FACS detection for the activity of indicated signaling pathways. *n* = 4 independent experiments for each detection. **B** *mcPGK1* overexpressing (oe*mcPGK1*) and control (oeVec) sample #2 cells were used for sphere formation, supplemented with Wnt/β-catenin inhibitors Wiki4 and LF3. Typical sphere images and calculated ratios were shown. Scale bars, 500 μm. **C** Immunoblot to evaluate the activation of Wnt/β-catenin signaling pathway in indicated treated sample #2 cells. **D** Immunofluorescent staining to detect the expression levels of TIC marker CD133 and Wnt/β-catenin target gene CCND1 in lactate and DM-αKG treated spheres, which were from sample #2 cells. Typical immunofluorescent images and statistical analysis of *n* = 10 fields were shown. Scale bars, 10 μm. **E** β-CATENIN, c-MYC and Ki67 immunohistochemistry in FX-11 treated, DM-αKG treated and control tumors. *n* = 6 tumors were detected per group. Typical β-CATENIN (β-CAT) immunohistochemistry results were shown. Scale bars, 20 μm. **F** Quantitative real-time PCR to detect the expression of Wnt/β-catenin-related genes in lactic acid and α-KG treated sample #2 cells. *n* = 4 independent experiments. **G** Western blot for β-catenin levels in cycloheximide (ChX) treated cells, which were cultured in 10 mM lactic acid or control medium. β-catenin levels were normalized to its level at 0 min. **H** Quantitative real-time PCR for the enrichment of indicated regions of *CTNNB1* promoter in ChIP eluate, in which H3K4me3 antibody and DM α-KG treated sample #2 cells were used. **I** Click-it EU labeling assay to detect the nascent CTNNB1 (β-CATENIN mRNA) levels. In all panels, data are shown as mean + s.d. For **A**–**C**, **F**–**I**, *n* = 4 independent experiments. For **A**, **B**, **D**, **E**, **I**, *P < 0.05; **P < 0.01; ***P < 0.001; ****P < 0.0001, by two-tailed Student’s *T*-test. Source data are provided as a Source Data file.

We evaluated several signaling pathways and found that lactic acid and α-KG both targeted Wnt/β-catenin pathway, a central signaling pathway for liver TIC function (Fig. 5A). The enhanced sphere-formation capacity by *mcPGK1* was blocked upon Wnt/β-catenin inhibition with Wiki4 or LF3, further confirming that *mcPGK1* exerted its role via Wnt/β-catenin pathway (Fig. 5B and Supplementary Fig. 7B). Lactic acid promoted Wnt/β-catenin activation and α-KG inhibited Wnt/β-catenin activation (Fig. 5C–F). Interestingly, β-catenin was increased at protein level but not mRNA level upon lactic acid treatment, and decreased at mRNA level upon DM-αKG treatment (Fig. 5C, F). As expected, lactic acid promoted β-catenin protein stability (Fig. 5G), and α-KG inhibited the activation of *β-catenin* promoter and *β-catenin* transcription (Fig. 5H, I). We then generated β-catenin silenced cells, and revealed that *mcPGK1* had an impaired function in liver TIC self-renewal and in vivo propagation of liver tumor cells, confirming that *mcPGK1* exerted its role mainly via a β-catenin-dependent manner (Supplementary Fig. 7C, D).

We then explored the molecular mechanism of lactic acid regulation of β-catenin protein stability. We previously identified *lnc-β-catm* as a modulator as β-catenin stability in liver cancer and TICs, via promoting the methylation of β-catenin^35^, and here we investigated whether lactic acid promoted the expression of *lnc-β-catm*. We revealed that lactic acid promoted *lnc-β-catm* expression (Supplementary Fig. 7E), and subsequent β-catenin methylation (Supplementary Fig. 7F). Moreover, lactic acid displayed a limited role on β-catenin stability and sphere formation in *lnc-β-catm* knockout cells, indicating the critical role of *lnc-β-catm* in lactic acid-driven β-catenin stability (Supplementary Fig. 7G, H). We then explored the molecular mechanism of lactic acid in *lnc-β-catm* expression. Considering the direct effect of lactic acid on histone lactylation^36^, we firstly detected the lactylation of *lnc-β-catm* promoter, and found that lactic acid promoted the histone lactylation of *lnc-β-catm* promoter at −700~−500 fragment (Supplementary Fig. 7I). We also deleted this region through CRISPR/Cas9 approach, and lactic acid didn’t promote *lnc-β-catm* expression upon *lnc-β-catm* promoter deletion (Supplementary Fig. 7J). These results proved that lactic acid promoted β-catenin stability largely through *lnc-β-catm* expression, which depends on histone lactylation of *lnc-β-catm* promoter.

Previous works have revealed that α-KG is a cofactor of H3K4me3 demethylase JARID1B^37,38^, thus we investigated whether α-KG regulates the transcription of β-catenin through JARID1B-mediated H3K4me3 modification. We found α-KG inhibited H3K4me3 levels (Supplementary Fig. 8A). Moreover, α-KG inhibited the chromatin accessibility at *CTNNB1* (β-catenin mRNA) promoter (Supplementary Fig. 8B). We also generated JARID1B silenced cells, and revealed that α-KG showed impaired roles in the H3K4me3 and accessibility of *CTNNB1* promoter, as well as nascent *CTNNB1* mRNA expression, upon JARID1B knockdown, indicating that α-KG inhibited β-catenin transcription through H3K4me3 demethylase JARID1B (Supplementary Fig. 8C–E). We also evaluated the function of α-KG with 2-HG, a competitive inhibitor of α-KG-dependent dioxygenases^38^, and found that 2-HG largely attenuated the functions of α-KG in H3K4me3 enrichment, accessibility of *CTNNB1* promoter and nascent *CTNNB1* mRNA, further confirming that α-KG functions as a modulator of H3K4me3 demethylase (Supplementary Fig. 8F–H). These findings indicate that both lactic acid and α-KG function in liver TICs through Wnt/β-catenin pathway.

### *McPGK1* interacts with PGK1

**Fig. 6:**
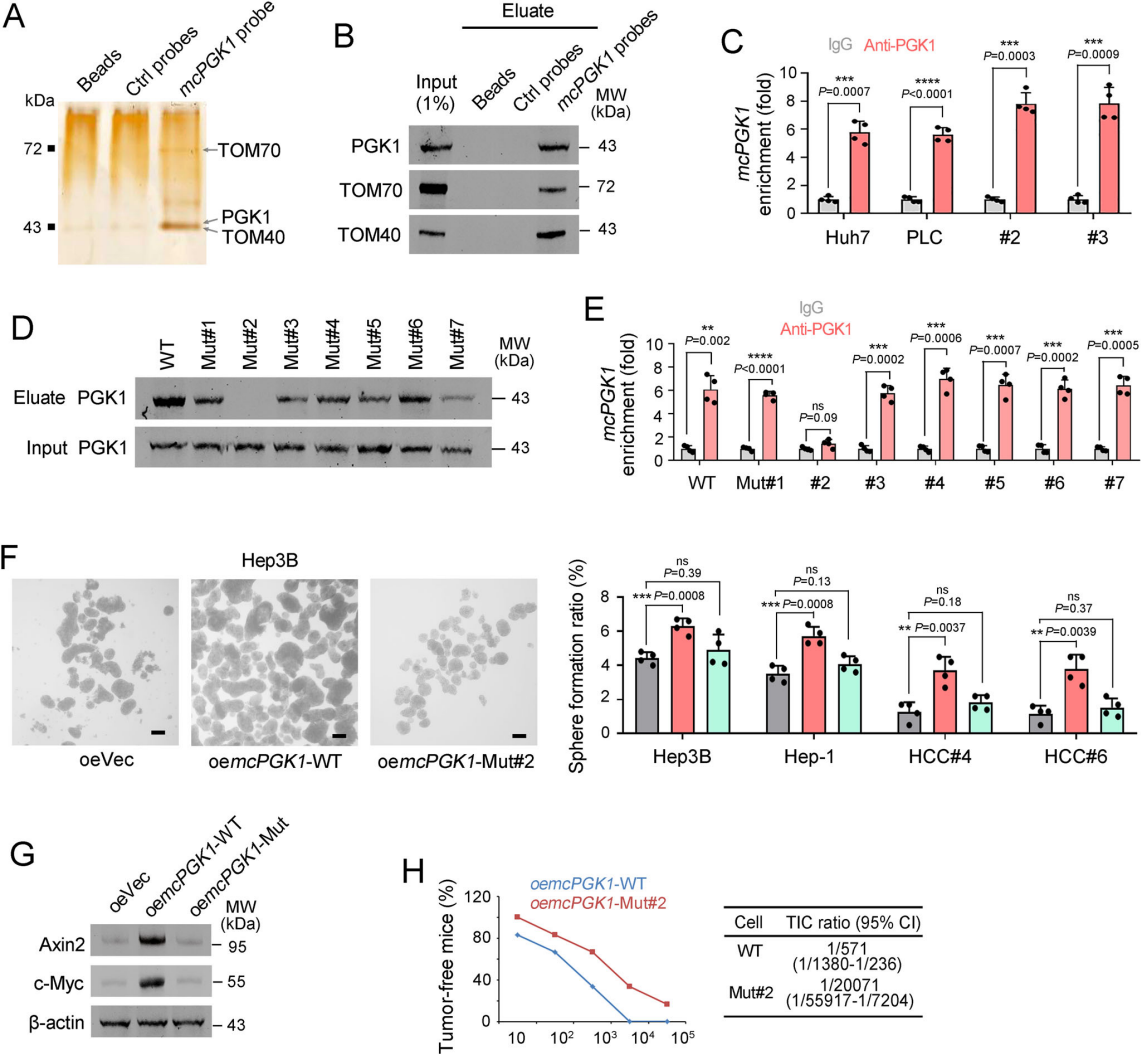
*McPGK1* functions as PGK1 partner. **A** Silver staining of RNA pulldown eluate, for which *mcPGK1* probes, control probes, and Huh7 sphere lysate were used. The specific bands indicated by black arrows were identified as PGK1, TOM70 and TOM40 via mass spectrum. **B** Immunoblot of RNA pulldown eluate to confirm the interaction of *mcPGK1* with PGK1, TOM70 and TOM40. Huh7 spheres were used for RNA pulldown. **C** Quantitative real-time PCR for *mcPGK1* enrichment in eluate sample from RNA immunoprecipitation, in which IgG and PGK1 antibodies were used. *mcPGK1* enrichment levels were normalized to IgG group. **D** WT and indicated mutant *mcPGK1* were used for TRAP assay, and the enrichment of PGK1 in TRAP eluate was evaluated through immunoblot. **E** Quantitative real-time PCR for the enrichment of *mcPGK1* in PGK1 RIP eluate. For RIP, *mcPGK1* lowly expressed cells (Hep3B) were overexpressed with indicated mutant *mcPGK1* transcripts. **F** Sphere formation of indicated Hep3B cells. *mcPGK1*-WT, wide type *mcPGK1*; *mcPGK1*-Mut#2, *mcPGK1* mutation losing the second stem-loop region. Scale bars, 500 μm. **G** Immunoblot to detect the activation of Wnt/β-catenin signaling in Hep3B cells overexpressing WT *mcPGK1* and mutant#2 *mcPGK1*. β-actin is a loading control. **H** The ratios of tumor-free mice initiated from indicated cells were shown in left panel, TICs ratios were shown in right panel. *n* = 6 6-week-old male BALB/c nude mice per group. For all panels, *n* = 4 independent experiments, and data are shown as mean + s.d. For **C**, **E**, **F**, **P < 0.01; ***P < 0.001; ****P < 0.0001; ns, not significant, by two-tailed Student’s *T*-test. Source data are provided as a Source Data file.

To analyze the molecular mechanisms of *mcPGK1*, we performed an RNA pulldown assay, which identified PGK1, TOM40 and TOM70 as the partners of *mcPGK1* in liver TICs (Fig. 6A). Immunoblot assay confirmed that *mcPGK1* interacted with PGK1, TOM40 and TOM70 (Fig. 6B). We focused on PGK1, which is a critical modulator in glycolysis and OXPHOS^39^. RNA immunoprecipitation proved PGK1-*mcPGK1* interaction (Fig. 6C). We further analyzed the interaction of *mcPGK1* and PGK1. Considering the critical role of stem-loop structures in RNA–protein interactions^40,41^, we analyzed the structure of *mcPGK1* and identified seven loops, and found that the second loop (HR#2) was required for the interaction between *mcPGK1* and PGK1 (Fig. 6D, E, and Supplementary Fig. 8I). These results confirmed that *mcPGK1* interacts with PGK1 in liver TICs.

We then examined whether the interaction with PGK1 was necessary for *mcPGK1*’s function. We generated cells that were overexpressing mutant *mcPGK1*, which lost the ability to interact with PGK1. Compared with wild-type *mcPGK1*, mutant *mcPGK1* did not promote Wnt/β-catenin activation and liver TIC maintenance, further confirming the essential role of PGK1-*mcPGK1* interaction (Fig. 6F, G). Moreover, mutant *mcPGK1* showed an impaired role in tumor initiation (Fig. 6H). Altogether, *mcPGK1* interacts with PGK1 and functions through *mcPGK1*-PGK1 interaction.

### *McPGK1* promotes the interaction of PGK1 and TOM40 complex

**Fig. 7:**
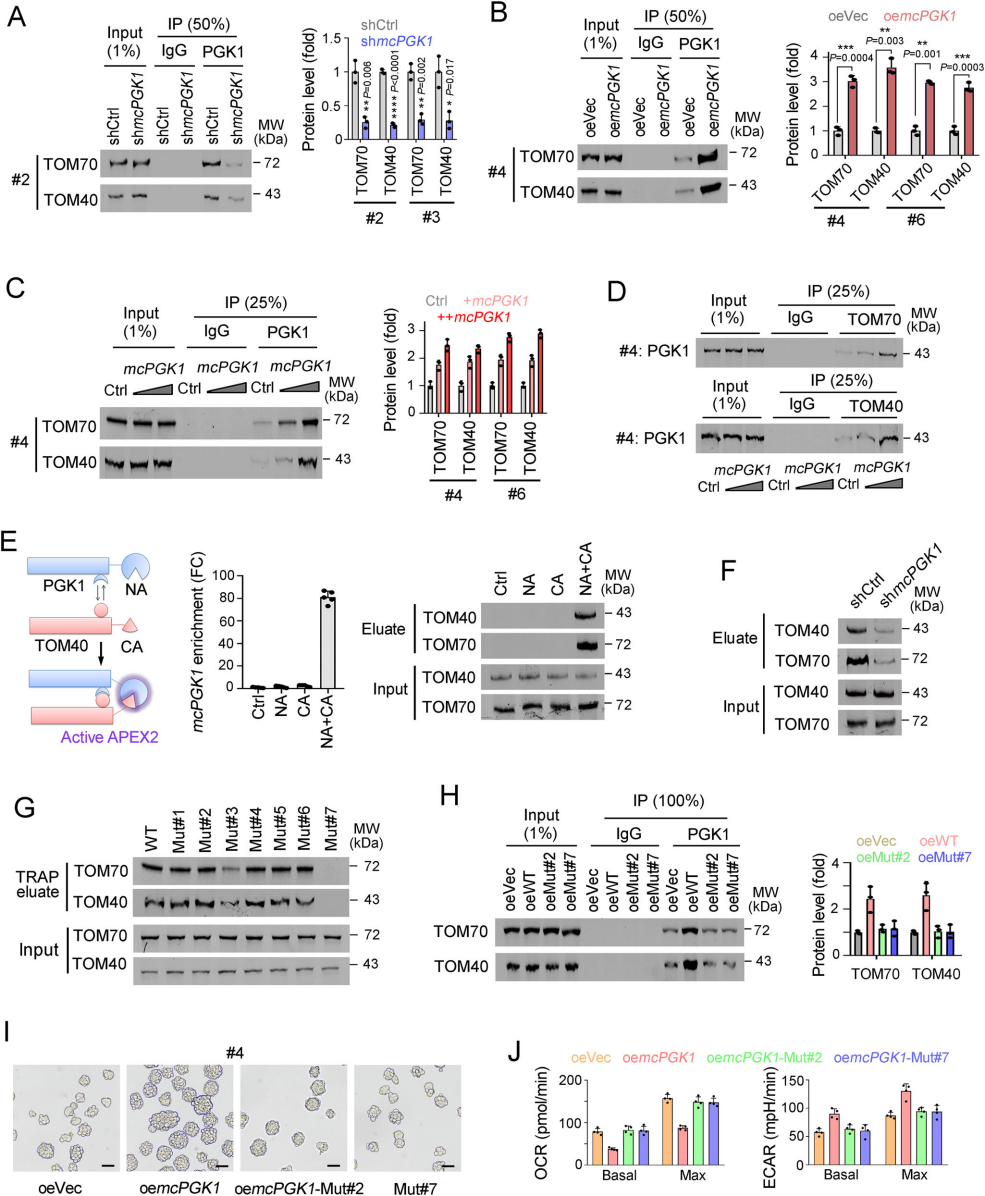
*McPGK1* promotes the binding of PGK1 and TOM40 complex. **A**, **B** Immunoblot of TOM70/TOM40 in PGK1 immunoprecipitation (IP) eluate from *mcPGK1* knockdown (**A**) or overexpressing (**B**) sphere lysate. 1% Input, 50% IgG IP eluate and PGK1 IP eluate were used for immunoblot. Typical results in left panel and protein quantitative results in right panel. **C**, **D** Immunoblot to evaluate TOM70/TOM40 levels in PGK1 IP eluate (**C**), or PGK1 levels in TOM70/TOM40 IP eluate (**D**). Primary #4 and #6 cells were used for sphere formation, and sphere lysates supplemented with gradient doses of *mcPGK1* transcript were used for IP assay. **E** Split-APEX2 assay were established (left), followed by real-time PCR for *mcPGK1* detection (middle) and Western blot for another outer mitochondrial membrane protein TOM70 (right). TOM40, PGK1 and *mcPGK1* are assembled together in outer mitochondrial membrane. **F** *mcPGK1* silenced cells were used for Split-APEX2, followed by Western blot with TOM40 and TOM70 antibodies. **G** Hep3B cells overexpressing WT and indicated mutant *mcPGK1* were used for TRAP assay, and the enrichment of TOM70/TOM40 in TRAP eluate was evaluated via immunoblot. **H** Immunoblot of TOM70/TOM40 in PGK1 IP eluate. WT and mutant *mcPGK1* cells were used for sphere formation, followed by IP assay with IgG control or PGK1 antibodies. *n* = 3 independent experiments. **I**, **J** Liver tumor cells (sample #4) overexpressing WT and mutant *mcPGK1* were used for sphere formation (**I**) and metabolic analysis ( **J**). Scale bars, 500 μm. For **A**–**D**, *n* = 3 independent experiments; for **E**, *n* = 5 independent experiments; for **I**, **J**, *n* = 4 independent experiments. For **D**, **F**, **G**, *n* = 3 independent experiments with similar results. Data are shown as mean + s.d. *P < 0.05; **P < 0.01; ***P < 0.001, by two-tailed Student’s *T*-test. Source data are provided as a Source Data file.

We then evaluated the combination between PGK1 and TOM40/TOM70, which are core components of TOM40 mitochondria importing complex^42^. We found that PGK1 interacted with TOM40 and TOM70, and their interactions were impaired in *mcPGK1* silenced cells (Fig. 7A). On the contrary, enhanced interactions between PGK1 and TOM40/TOM70 were detected in *mcPGK1* overexpressing cells (Fig. 7B). Enhanced interactions between PGK1 and TOM40/TOM70 were confirmed by co-immunoprecipitation assay supplemented with gradient *mcPGK1* (Fig. 7C, D). Split-APEX2 assay confirmed the assembly of PGK1-TOM40/TOM70-*mcPGK1* complex at outer mitochondrial membrane (Fig. 7E). Moreover, attenuated assembly of PGK1-TOM40/TOM70 complex was detected upon *mcPGK1* knockdown (Fig. 7F). These results demonstrated that *mcPGK1* promoted the interaction between PGK1 and TOM40/TOM70. Using mutant *mcPGK1* transcripts, we found that the seventh loop (HR#7) was required for the interaction between *mcPGK1* and TOM40/TOM70 (Fig. 7G). HR#2 mutant and HR#7 mutant *mcPGK1* transcripts were overexpressed, which lost the ability to interact with PGK1 and TOM40/TOM70, respectively. Both mutant *mcPGK1* transcripts (mut#2 and mut#7) weren’t involved in the regulation of PGK1-TOM40/TOM70 interaction, indicating that *mcPGK1* served as a scaffold of PGK1-*mcPGK1*-TOM40/TOM70 complex (Fig. 7H). Moreover, #2 mutant and #7 mutant *mcPGK1* weren’t involved in liver TIC self-renewal and metabolic reprogramming (Fig. 7I, J). HR#2 and HR#7 mutant mcGPK1 transcripts had impaired roles in PGK1-TOM40 interaction, TIC self-renewal and metabolic reprogramming, whereas WT mcGPK1 displayed these roles, thus we concluded that HR#2 and HR#7 were required for PGK1-TOM40/TOM70 interaction and *mcPGK1*-driven TIC self-renewal. Altogether, *mcPGK1* promotes the binding of PGK1 to TOM40 mitochondrial importing complex.

### *McPGK1* drives the mitochondrial entry of PGK1

**Fig. 8:**
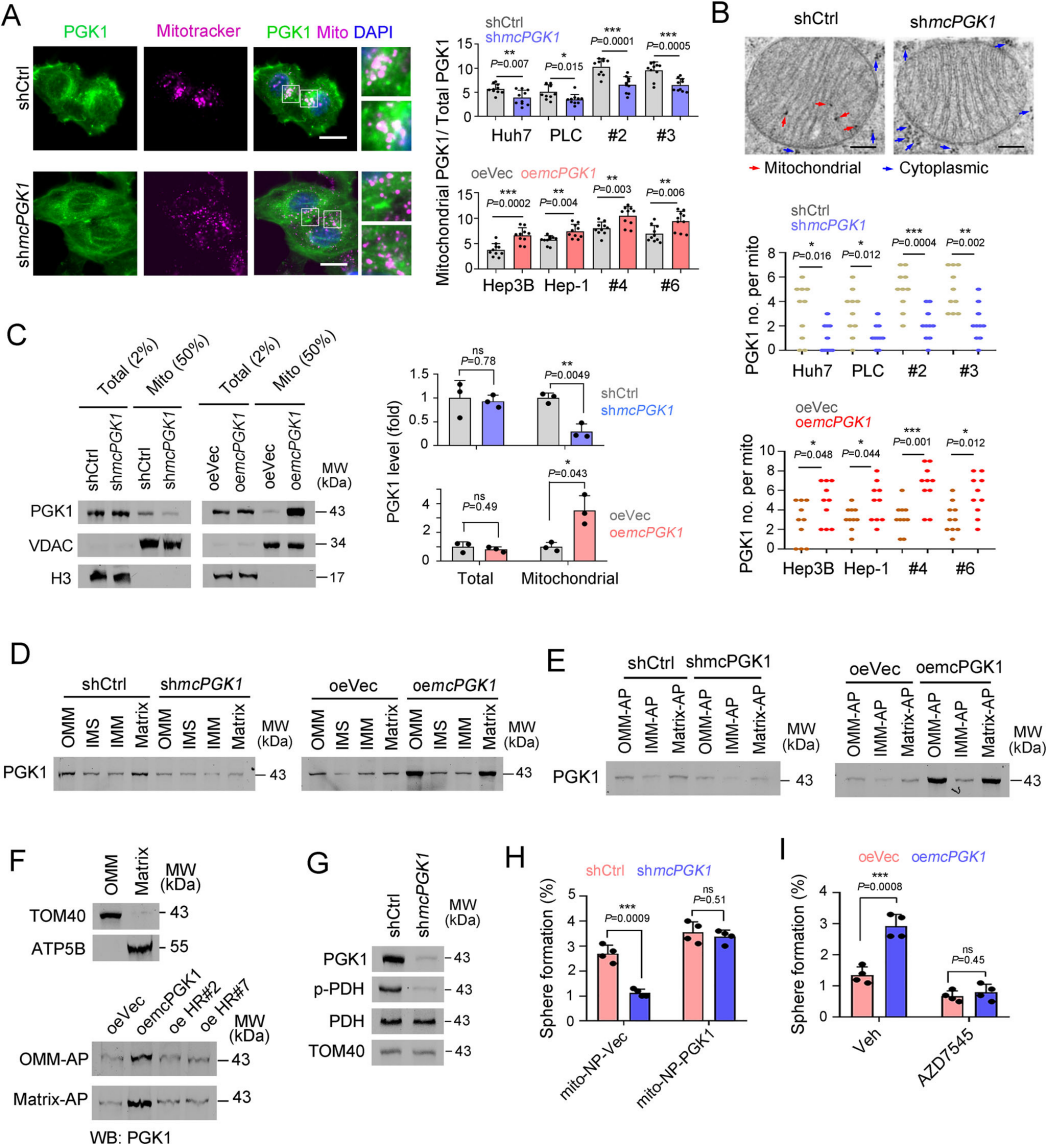
*McPGK1* promotes the mitochondrial entry of PGK1. **A** Confocal microscopy to detect the intracellular location of PGK1 in *mcPGK1* silenced and control cells. Scale bars, 10 μm. *n* = 10 fields per group. **B** Immuno-electron microscopy for the mitochondrial entry of PGK1. *n* = 5 independent experiments with similar results (upper panel) and *n* = 10 mitochondria images for statistical analysis (lower panel). Scale bars, 200 nm. **C** Immunoblots for the mitochondrial entry of PGK1. Voltage-dependent anion channel (VDAC) (mitochondrial) and histone 3 (H3) (nuclear) were used to detect the purity of mitochondria. Huh7 cells were used for *mcPGK1* knockdown and Hep3B cells were used for *mcPGK1* overexpression. *n* = 3 independent experiments. **D** The indicted fractions of mitochondria were isolated and PGK1 levels were detected with immunoblot. **E** OMM-APEX, IMM-APEX and matrix-APEX were established in mcPGK1 silenced and overexpressed cells, followed by streptavidin pulldown and PGK1 immunoblot was performed to detect the enrichment of PGK1 in mitochondria. **F** Hep3B cells expressing OMM-APEX (OMM-AP) system for outer mitochondrial membrane labeling, or expressing Matrix-APEX (Matrix-AP) system for mitochondrial matrix labeling, were used for *mcPGK1* or mutant (HR#2, HR#7) *mcPGK1* overexpression. **G** Immunoblot for PDH and PDH inactivating phosphorylation (p-PDH1) levels in mitochondrial fractions, which were isolated from the indicated cells. **H** PGK1 mitochondria-locating #2 cells were established via mitochondria-targeting nanoparticle (mito-NP) and then *mcPGK1* was silenced, followed by sphere formation assay. *n* = 4 independent experiments. **I** Sphere formation of *mcPGK1* overexpressing (oe*mcPGK1*) and control (oeVec) sample #4 cells, supplemented with 100 nM PDK1 inhibitor AZD7545. *n* = 4 independent experiments. For **D**–**G**, *n* = 3 independent experiments with similar results. In all panels, data are shown as mean + s.d. *P < 0.05; **P < 0.01; ***P < 0.001, ns, not significant, by two-tailed Student’s *T*-test. Source data are provided as a Source Data file.

PGK1 is expressed in the cytoplasm, but is often translocated to the mitochondria during tumorigenesis, where it phosphorylates PDK1 at T338. In turn, PDK1 phosphorylates and inhibits the PDH complex, and thus inhibits OXPHOS and promotes glycolysis^43^. Here, we demonstrated that *mcPGK1* promoted the binding of PGK1 to TOM40 mitochondria importing complex. Therefore we examined the involvement of *mcPGK1* in the translocation of PGK1 to mitochondria and found that this translocation was suppressed in *mcPGK1* silenced cells, and increased in *mcPGK1* overexpressing cells (Fig. 8A). However, PGK1 expression wasn’t influenced by *mcPGK1* (Supplementary Fig. 9A). Immuno-electron microscopy also revealed that *mcPGK1* was involved in mitochondrial translocation of PGK1 (Fig. 8B). Mitochondrial isolation and immunoblot confirmed that *mcPGK1* was essential for PGK1 mitochondrial translocation (Fig. 8C). The positive role of *mcPGK1* in PGK1 mitochondrial entry was further confirmed by mitochondrial fraction separation assay (Fig. 8D, E). We then overexpressed WT and mutant *mcPGK1* transcripts and detected the mitochondrial translocation of PGK1 via Matrix-APEX and OMM-APEX. Overexpression of WT *mcPGK1* promoted the mitochondrial translocation of PGK1, whereas HR#2 and HR#7 mutant transcripts had no such effect, further confirming the essential role of HR#2/#7 in *mcPGK1*-dependent mitochondrial translocation of PGK1 (Fig. 8F).

We then examined and found that *mcPGK1* knockdown decreased the inactivating phosphorylation of PDH (Fig. 8G). As expected, mcPGK1-PGK1-PDK1 mediated PDH phosphorylation inhibited PDH’s function of converting pyruvate to acetyl-CoA, and subsequently drove a metabolic reprogramming from OXPHOS to glycolysis (Supplementary Fig. 9B). These data confirmed the role of mcPGK1-PGK1-PDK1-PDH axis in metabolism reprogramming from OXPHOS to glycolysis (Supplementary Fig. 9C). Finally we evaluated the role of PGK1-PDK1-PDH axis in *mcPGK1* function. Nanoparticle-delivered PGK1 mitochondrial translocation largely diminished the role of *mcPGK1* knockdown, indicating that *mcPGK1* functions through PGK1 mitochondria-entry (Fig. 8H and Supplementary Fig. 9D). Moreover, *mcPGK1* overexpression had a limited role in sphere formation upon PDK1 blockade (Fig. 8I). Similarly, *mcPGK1* overexpression also showed impaired roles in sphere formation and in vivo propagation upon PDK1 was silenced, further confirming that *mcPGK1* exerted its functions through PDK1-PDH pathway (Supplementary Fig. 9E, F). Taken together, these results indicate that *mcPGK1* drives mitochondrial translocation of PGK1, inhibits OXPHOS and promotes glycolysis via the PGK1–PDK1–PDH pathway.

## Discussion

In this work, we identified mitochondria-encoded *mcPGK1* promotes liver TIC self-renewal via metabolic reprogramming from OXPHOS to glycolysis. *McPGK1* interacts with PGK1, promotes the binding of PGK1 to TOM40 mitochondrial importing complex, and drives the mitochondrial translocation of PGK1. Metabolic reprogramming switches the metabolites from α-KG to lactic acid, activates Wnt/β-catenin and liver TIC function. Our work reveals an additional layers to circRNA function, TIC self-renewal and metabolism regulation.

circRNAs, generated by back-splicing of the 3' and 5' ends of RNA, have emerged as critical modulators in a variety of biological processes. With recent advances in RNA sequencing, several new types of circRNAs have been identified, including read-through, virus-encoded, and mitochondria-encoded circRNAs^22^. Here, we focus on mitochondria-encoded circRNA in liver TICs. Mitochondria contain a copy of circular double-stranded DNA, about 16.5 kb long, from which circRNAs are generated. Very recently, the mitochondria-encoded circRNA *SCAR* was discovered to alleviate NASH by reducing mROS output^23^. Here, we identified TIC-regulatory function of *mcPGK1*, a newly identified mitochondria-encoded circRNA, adding an additional layer to the function of circRNAs and regulation of TICs.

Mitochondria contain 1000–3000 proteins, most of which are encoded by nuclear genes. The blockade of mitochondrial translocation triggers various disorders, including obesity^44^. Here we revealed that a dysregulated mitochondrial translocation of PGK1 drives liver tumorigenesis, TIC self-renewal and metabolic reprogramming. Because each cell contains many copies of mitochondrial DNA, it is difficult to manipulate the expression of mitochondrial genes, which hinders research aimed at investigating their biological roles. Several studies have revealed that siRNA and shRNA can be used to silence gene expression in mitochondria^45^, and a CRISPR-free mitochondrial base editing system has been created that can change C:G to T:A in mitochondrial DNA in an efficient and specific manner^46^. A mitochondria-targeting nanoparticle has also been constructed and used to deliver genes to mitochondria^23^. In this study, we used shRNA to silence *mcPGK1* expression. Nuclear overexpression of *mcPGK1* was implicated in liver TIC self-renewal and metabolic reprogramming. Indeed, while *mcPGK1* is mainly localized to the mitochondria, it is also present in the cytoplasm. We also demonstrated that *mcPGK1* was required for the mitochondrial translocation of PGK1, which was normally localized to the cytoplasm.

Mitochondrial metabolism is closely related to stemness regulation. Actually, increased mitochondrial biogenesis and OXPHOS induce the differentiation of various stem cells, indicating that the loss of mitochondrial function is very important for stemness maintenance^47^. Mitochondrial biogenesis, fission and metabolic plasticity are involved in asymmetric division and prostate TIC self-renewal^48^. Some stemness factors, such as Nanog, reduce OXPHOS activity and decrease mROS production to maintain the self-renewal capacity^49^. Here, we revealed that in liver TICs, a highly expressed circRNA drives metabolic reprogramming from OXPHOS to glycolysis by modulating the mitochondrial distribution of PGK1. Metabolic reprogramming from OXPHOS to glycolysis may provide a material basis for the rapid propagation of tumor cells, and the metabolites may also play key roles in the regulation of stemness. Indeed, here we found that lactic acid and α-KG are involved in Wnt/β-catenin activation and liver TIC function.

In addition to TIC self-renewal, metabolic reprogramming contributes to drug resistance and immune escape. The reduction in OXPHOS results in reduced mROS production, promotes a cellular quiescent state, and maintains the genomic stability of stem cells^50^. The reduction in mROS production via metabolic reprogramming in TICs is thought to play a key role in resistance to chemotherapeutic drugs^51^. Glycolysis also inhibits anti-tumor immune activity^52^. In particular, TICs increase the production of lactic acid through glycolysis, thus maintaining an acidic tumor microenvironment that inhibits the function of anti-tumor immune cells such as T effector and natural killer cells^53^. Therefore, the metabolic reprogramming from OXPHOS to glycolysis may inhibit immune surveillance during tumorigenesis. Hence, *mcPGK1* might contribute to tumor immune escape and the therapeutic effect of immune checkpoint therapy of liver tumors.

## Methods

### Ethics statement, mice and cells

This work was approved by the ethics committee of Zhengzhou University (ZZUIRB202054 and ZZUIRB202055). For all mouse experiments, 6-week-old male BALB/c nude mice were purchased were purchased from Beijing Vital River Laboratory Animal Technology Co., Ltd., and housed in the animal facility at School of Life Sciences, Zhengzhou University. Mice were housed in SPF condition, with 4-7 mice per cage in 12 h light/dark cycle (7:00-19:00 light, 19:00-7:00 dark), with controlled room temperature (23 ± 2 °C) and humidity (40-60%). All mice were randomly grouped and no mice were excluded from analyses. The maximal tumor burden over 2500 mm^3^ is forbidden by the ethics committee, and this limit was not exceeded in all experiments. All efforts to minimize animal suffering were made. Liver cancer tissues used in this work were obtained from The First Affiliated Hospital of Zhengzhou University. Hep3B cells were obtained from ATCC (catalog no, HB-8064), Huh7 cells were obtained from iCellbioscience (catalog no. iCell-h080), 293 T, PLC and Hep-1 cells were from Zusen Fan lab (Institute of Biophysics, Chinese Academy of Sciences).

### Antibodies and Reagents

Anti-β-Catenin (catalog no. 610153) and anti-CD133 antibody (catalog no. 566598) was purchased from BD Bioscience. Anti-PGK1 (catalog no. 68540 S), anti-EEA1 (catalog no. 3288 S), anti-β-actin (catalog no. 4970), anti-H3 (catalog no. 4499) and anti-H3K4me3 (catalog no. 9751 S) antibodies were from Cell Signaling Technology. Anti-ZIC2 (catalog no. ARP35821_P050) antibody was purchased from Aviva Systems Biology. Anti-TOM40 (catalog no. 18409-1-AP), anti-TOM70 (catalog no. 14528-1-AP), anti-c-MYC (catalog no. 10828-1-AP) and anti-AXIN2 (catalog no. 20540-1-AP) antibodies were from Proteintech Group, Inc. Goat anti-Mouse IgG (H + L) Cross-Adsorbed Secondary Antibody, Alexa Fluor™ 594 antibody (catalog no. A-11005), Goat anti-Rabbit IgG (H + L) Cross-Adsorbed Secondary Antibody, Alexa Fluor™ 488 (catalog no. A-11008), Goat anti-Rabbit IgG (H + L) Cross-Adsorbed Secondary Antibody, Alexa Fluor™ 647 (catalog no. A-21244) were purchased from Invitrogen. HRP-conjugated Affinipure Goat Anti-Mouse IgG(H + L) antibody (catalog no. SA00001-2) and HRP-conjugated Affinipure Goat Anti-Rabbit IgG(H + L) antibody (catalog no. SA00001-2) were purchased from Proteintech Group, Inc. Polymer HRP and AP detection kits were from Beyotime Biotechnology. Biotin labeled RNA mix (catalog no. 11685597910) was from Roche.

### Tumor initiation assay

For tumor initiation assay, 10, 1 × 10^2^, 1 × 10^3^, 1 × 10^4^, and 1 × 10^5^ *mcPGK1* knockdown, overexpressing cells were subcutaneously transplantated into 6-week-old male BALB/c nude mice and tumor initiation was detected after 3 months. Online-available Extreme Limiting Dilution Analysis tool (http://bioinf.wehi.edu.au/software/elda/)^54^ was used for TIC ratio calculation.

### Sphere formation

For sphere formation, 1000 Huh7 and PLC single cells were cultured in Ultra Low Attachment 6-well plates, and incubated with Dulbecco’s modified Eagle’s medium/F12 (Life Technologies) supplemented with N2, B27, 20 ng/ml EGF and 20 ng/ml bFGF (Millipore) for 2 weeks, sphere initiating ratio = (sphere number)/1000 × 100%. For primary cells, 5000 cells were used for each well, and sphere initiating ratio = (sphere number)/5000 × 100%.

### Separation of mitochondria and mitochondrial fractions

*mcPGK1* silenced, overexpressing and control cells were harvested for mitochondria isolation. Isolation buffer (225 mM mannitol, 20 mM MOPS, 75 mM sucrose, 1 mM EGTA, 0.1% BSA, pH 7.2) and lysis buffer (100 mM sucrose, 10 mM MOPS, 1 mM EGTA, 0.1% BSA, pH 7.2) were used for mitochondria isolation.

For separation of mitochondrial fractions, mitochondria were incubated with 1 ml digitonin buffer (mitochondria isolation buffer containing 0.5 mg/ml digitonin) for 15 min, followed by 10,000 x g (10 min, 4 °C) centrifuge. The pellet contained mitoplast and the supernatant contained OMM and IMS fractions. Then OMM fraction was obtained from the precipitate of 10,000 x g (30 min, 4 °C) centrifuge. The mitoplast pellet was re-suspended into 0.2 ml mitochondria isolation buffer and gently disrupted with ultrasonication, followed by 10,000 x g (30 min, 4 °C) centrifuge. The IMM fraction was in pellet and matrix fraction was in supernatant.

### Absolute quantification of *mcPGK1*

For absolute quantification, total cells or cell fractions from peri-tumor, tumor, TIC and sphere cells were used for RNA extraction, followed by 1U/mg RNase R treatment at 37 °C for 15 min. RNA samples were then reversely transcribed to cDNA and quantitative real-time PCR was performed. In vitro transcribed *mcPGK1* was used for standard curve by series dilution.

### APEX submitochondrial fractions

For APEX assay, liver cancer cells were transfected with Matrix-APEX2 (Cat# 72480, Addgene)^55^, IMS-APEX2 (Cat# 79058, Addgene)^56^, or OMM-APEX2 plasmids (Cat# 79056, Addgene)^55^ for submitochondrial labeling. The cells were treated with 500 mM biotin-phenol at 37 °C for 30 min, and then treated with 1 mM H_2_O_2_ at room temperature for 1 min. Samples were then treated with 2 mL azide-free quenching solution and 5 mM Trolox for 1 min. Streptavidin-conjugated magnetic beads were washed twice with RIPA lysis buffer (150 mM NaCl, 1% NP-40, 0.5% sodium deoxycholate, 0.1% SDS, 1 mM EDTA, 50 mM Tris, pH 8.0), and subjected into whole cell lysate for 2 h incubation. After washing with RIPA buffer four times, beads were boiled for 15 min and subjected into Western blot.

### Preparation of Mito-nanoparticle

The mito-nanoparticles were designed and synthesized as discribed^23,57^. Mitochondria-targeting peptide was synthesized by CHENPEPTIDE Biotechnology Co Ltd (Nanjing, China). PSiCoR (Cat# 12084, Addgene) was used for shRNA expression, and modified PCDNA4 plasmid was used for *mcPGK1* overexpression. The sequence of the *mcPGK1* shRNA and overexpression was confirmed by Sanger sequencing.

### Lentivirus generation and cell infection

pSiCoR was used for knockdown. Sequences of shRNAs targeting the junction sequence of *mcPGK1* were cloned into pSiCoR vector (Cat no. 12084, Addgene). For lentivirus packaging, we transfected 293 T cells with pSiCoR and package plasmids (4 mg pSiCoR vector, 1 mg VSVG, 1 mg RRE and 2 mg RSV-REV were used for 10 cm dish). PLC, Huh7 and HCC primary cells were infected by virus supernatants or PEG5000 (Sigma)-enriched precipitates. *mcPGK1* overexpressing cells were established similarly. shRNA sequences for PGK1, PDK1 and PDH used in this study were listed in Supplementary Table 1.

### RNA extraction and RT-PCR analyses

Total RNA samples were isolated with TRIzol method. 1 μg RNAs were reverse-transcribed into cDNA and then subjected to quantitative real-time PCR analysis with ABI QuantStudio5 Q5. Relative changes in expression levels were calculated. RT-qPCR primers are listed in Supplementary Table 2.

### RNA pulldown

Spheres were crushed with RIPA buffer supplemented with protease inhibitor cocktail and RNase inhibitor, and pre-cleared with streptavidin beads for 1 h. Biotin labeled RNA probes and cell lysis were mixed together in 4 °C for 3 h, and biotin-enriched components were separated and the binding proteins were detected with silver staining or immunoblot.

### Silver staining and mass spectrometry analysis

Pulldown samples from spheres by *mcPGK1* probes and antisense probes were boiled for 15 min, separated through 15% SDS-PAGE, and observed by sliver staining. The variant bands in *mcPGK1* eluate were identified through mass spectrometry analysis (LTQ Orbitrap XL).

### Immunoblot

For immunoblot, samples were crushed and boiled in 1×SDS-loading buffer for 15 min, and then proteins were separated by electrophoresis. Proteins were then transferred to nitrate cellulose (NC) membrane, followed by detection with primary antibody and HRP-conjugated antibodies, finally the HRP signals were visualized by ultra-sensitive enhanced chemiluminescent (ECL) substrate^58^.

### Northern blot

Total RNA from CD133^high^, CD133^low^, sphere and non-sphere samples was extracted with standard TRIzol method, separated with electrophoresis and transferred to positively charged NC film (Beyotime Biotechnology), and then cross-linked by UV exposure. RNA samples on NC membranes were detected with digoxin-labeled RNA probes, which were generated through in vitro transcription. Finally RNA signals were detected with HRP-conjugated anti-digoxin antibody.

### RNA immunoprecipitation

Spheres were lyzed in RNase-free RIPA buffer supplemented with RNase inhibitor and protease-inhibitor cocktail, centrifuged and supernatants were collected for preclear with Protein A/G. PGK1 and control antibodies were mixed with Protein A/G, followed by 4 h incubation with precleared sphere lysates. Finally RNA samples in eluate were extracted and *mcPGK1* enrichment was detected through quantitative real-time PCR.

### Signaling pathway activity reporter system

Wnt/β-catenin, Notch, Hedgehog, mTOR, NF-kB, P38, JNK, ERK and PKA reporter plasmids were overexpressed liver cancer cells, and treated with lactic acid or DM-αKG^59^. The activity levels of each signaling pathway were detected by FACS. For example, Wnt activity level = (TOP-GFP intensity)/(FOP-GFP intensity). The plasmids used in this assay are: TOP-GFP (addgene no. 35489), FOP-GFP (addgene no. 35490), 12XCSL-d1EGFP (addgene no. 47684), 7Gli:GFP (addgene no.110494), TORCAR (addgene no. 64927), NF-kB-eGFP (addgene no. 118093), TORCAR(T/A) (addgene no. 64928), p38KTRmCerulean3 (addgene no. 59155), JNKKTRmRuby2 (addgene no. 59154), PKAKTRClover (addgene no.59153), ERKKTRClover (addgene no. 59150).

### Reporting summary

Further information on research design is available in the Nature Portfolio Reporting Summary linked to this article.

## Supplementary information

Supplementary Information

Reporting Summary

## Data Availability

The circRNA sequencing data generated in this study have been deposited in the Gene Expression Omnibus (GEO) Database under accession code GSE223661. Source data generated in this study are provided in the Source Data file. Source data are provided with this paper.

## Source data

Source Data
