# Supplementary material for: mcPGK1-dependent mitochondrial import of PGK1 promotes metabolic reprogramming and self-renewal of liver TICs
Source: Nat Commun. 2023 Feb 27;14:1121. doi: 10.1038/s41467-023-36651-5 (PMC9971191; doi:10.1038/s41467-023-36651-5)
Supplement: Supplementary file 1 — Supplementary Information [file 41467_2023_36651_MOESM1_ESM.pdf]

Supplementary Information

mcPGK1-dependent mitochondrial import of PGK1 promotes metabolic reprogramming and self-renewal of liver TICs

Zhenzhen Chen[1, 6, *], Qiankun He[1,6], Tiankun Lu[1, 6], Jiayi Wu[2,6], Gaoli Shi[1], Luyun He[3], Hong Zong[5], Benyu Liu[4, *], Pingping Zhu [1,*]

1. School of Life Sciences, Zhengzhou University, 100 Kexue Road, Zhengzhou, Henan 450001, China.

2. School of Medicine, Nankai University, 94 Weijin Road, Tianjin, 300071, China

3. Department of Pathophysiology, School of Basic Medical Sciences, Zhengzhou University, Zhengzhou 450001, China.

4. Research Center of Basic Medicine，Academy of Medical Sciences，Zhengzhou University, Zhengzhou, Henan, China.

5. Department of Oncology, The First Affiliated Hospital of Zhengzhou University, NO.1 Eastern Jianshe Road, Zhengzhou, 450052, Henan, China.

6. These authors contributed equally to this work.

* Corresponding authors: Zhenzhen Chen (chenzz2015@zzu.edu.cn), Benyu Liu (benyuliu@zzu.edu.cn) and Pingping Zhu (zhup@zzu.edu.cn)

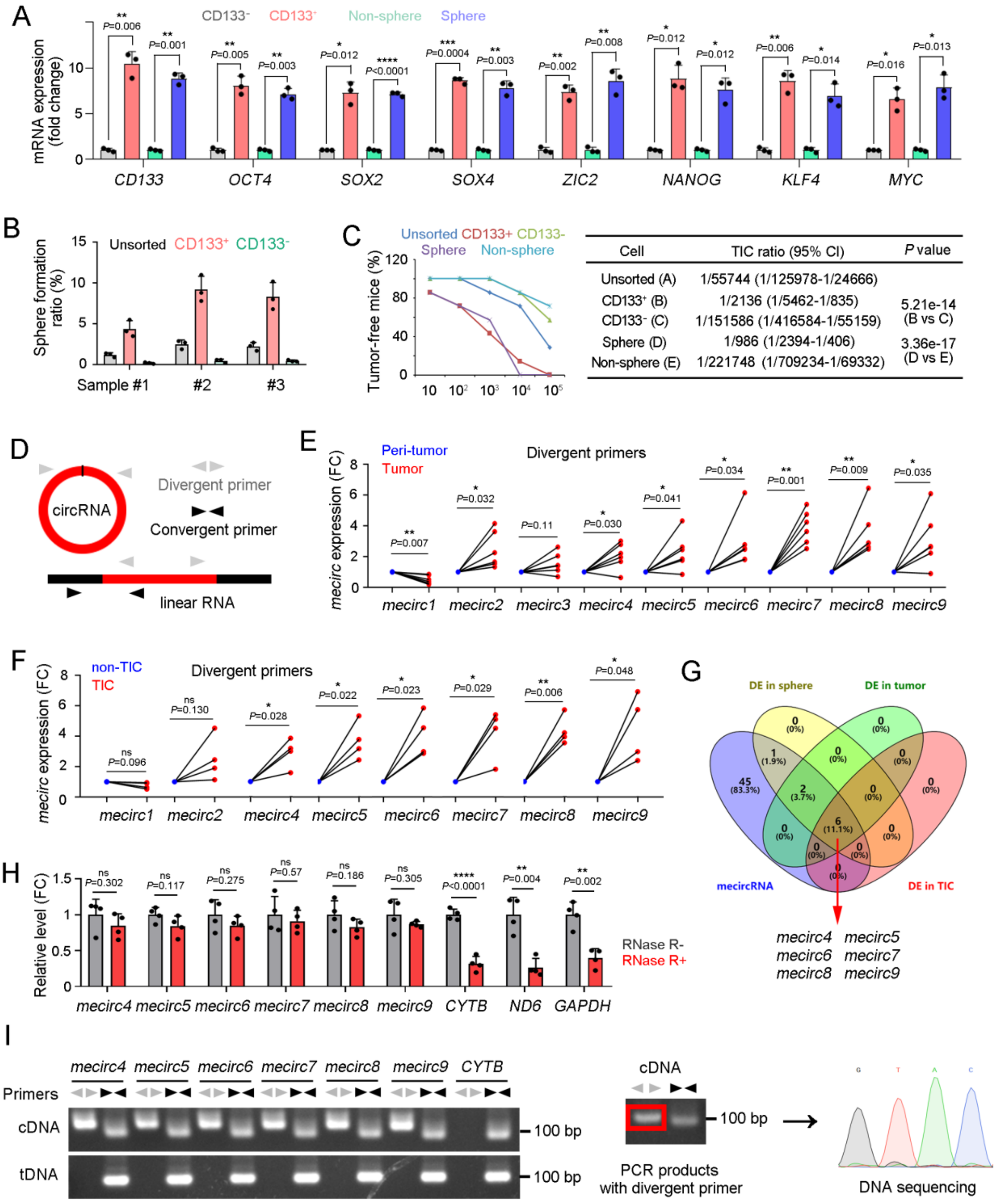

Supplementary Figure 1. The landscape of mecircRNA in liver cancer and TICs, related to Figure 1. (A) Quantitative real-time PCR analysis to detect the expression levels of TIC-associated genes in TICs ($CD133^+$), non-TICs ($CD133^-$), spheres and non-spheres. (B, C) Sphere formation (B) and tumour initiation (C) assays of $CD133^+$ and $CD133^-$ cells. For B, *n*=3 independent assays were performed and calculated ratios were shown. For C, *n*=7 6-week-old male BALB/c nude mice were subcutaneously injected with gradient numbers of the indicated cells for 3 months' tumour formation. TIC ratios and *P* values were calculated by extreme limiting dilution analysis (ELDA) (http://bioinf.wehi.edu.au/software/elda/). (D) Divergent primer and convergent primer were designed to specifically target circRNA and linear RNA, respectively. (E) Quantitative

real-time PCR analysis for mecircRNA expression in liver tumours and peri-tumours. Six pairs of peri-tumour and tumour samples were used for mecircRNA detection with divergent primers. Tumor expression levels were normalized to the corresponding peri-tumor samples. (F) TICs and paired non-TICs were used for mecircRNA detection and TIC levels were normalized to non-TIC levels. (G) Venn diagram showing the expression landscape of mecircRNAs in tumors, spheres and TICs. mecirc4, mecirc5, mecirc6, mecirc7, mecirc8 and mecirc9 were selected as their high expression in tumor cells, spheres and TICs. (H) Validation of the indicated mecircRNA as circRNA through RNase R treatment. Total RNAs extracted from liver TICs were treated with or without 3 U/g RNase R for 1 h. CYTB, ND6 and GAPDH serve as linear RNA control. n=4 independent experiments. (I) Confirmation of mecircRNA as circRNA with PCR and DNA sequencing. For PCR assay, complementary DNA (cDNA) and total DNA (tDNA) were used as templates to amplify mecircRNAs with divergent and convergent primers. In all panels, data are shown as mean + s.d. *$P < 0.05$; **$P < 0.01$; ***$P < 0.001$; ****$P < 0.0001$, by two-tailed Student's T-test. Source data are provided as a Source Data file.

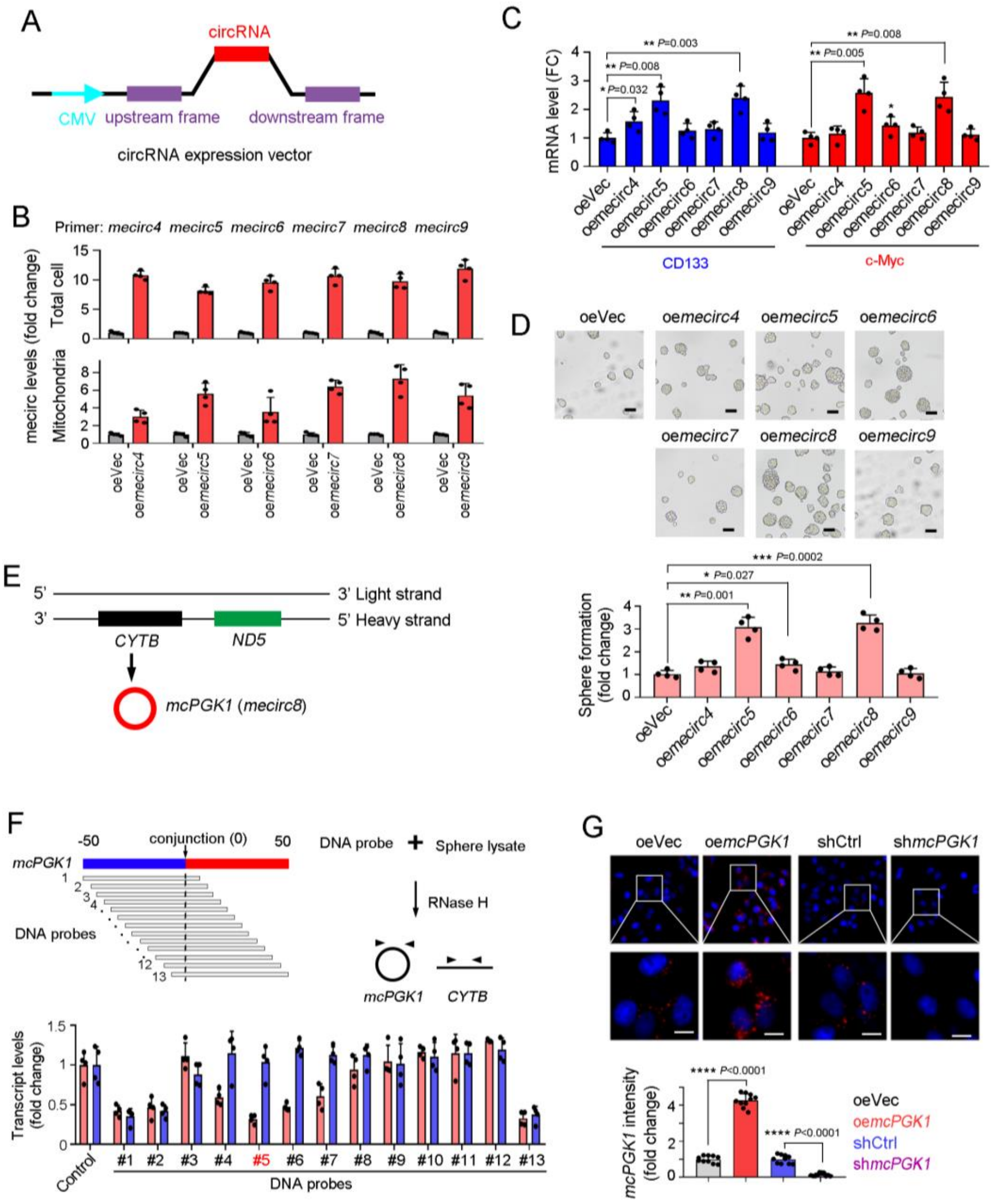

Supplementary Figure 2. Screening functional mecircRNA and characteristics of mcPGK1, related to Figure 1. (A) Schematic diagram showing the design strategy for circRNA expression. circRNA transcription was initiated by CMV promoter and circularization was driven by back-splicing of upstream frame and downstream frame. (B) mecircRNA overexpression cells (upper panel) and mitochondria (lower panel) were used for RNA extraction, and mecircRNA expression levels were examined through realtime PCR assay. All expression levels were normalized to control samples. (C) Expression levels of CD133 and c-Myc in mecircRNA overexpression and control cells were detected through quantitative real-time PCR. *n*=4 independent experiments, and all expression levels were normalized to those in control cells. (D) Sphere formation assays to detect self-renewal of the indicated cells. Typical images were shown in upper panels and sphere formation ratios were shown in lower panel. Scale bars, 500 μm. (E) Schematic diagram of mcPGK1. mcPGK1 was transcribed from *CYTB* loci of the heavy strand of

mitochondrial DNA. (F) Schematic diagram for mcPGK1 probe screening. Imbricate antisense DNA probes targeting the conjunction sequence of mcPGK1 were designed and incubated with sphere lysate, followed by 0.1 U/μl RNase H digestion for 30 min and RNA extraction. The levels of circular mcPGK1 and linear CYTB were analyzed by real-time PCR. The average levels of control probes of both transcripts were settled as 1. (G) The specificity of mcPGK1 probe was confirmed with mcPGK1 overexpressing (oemcPGK1) and knockdown (shmcPGK1) cells. Typical images were in the upper panel and calculated mcPGK1 intensities were in the lower panel. Scale bars, 10 μm. In all panels, *n*=4 independent experiments were performed with similar results, and data are shown as mean + s.d. **P* < 0.05; ***P* < 0.01; ****P* < 0.001; *****P* < 0.0001, by two-tailed Student's T-test. Source data are provided as a Source Data file.

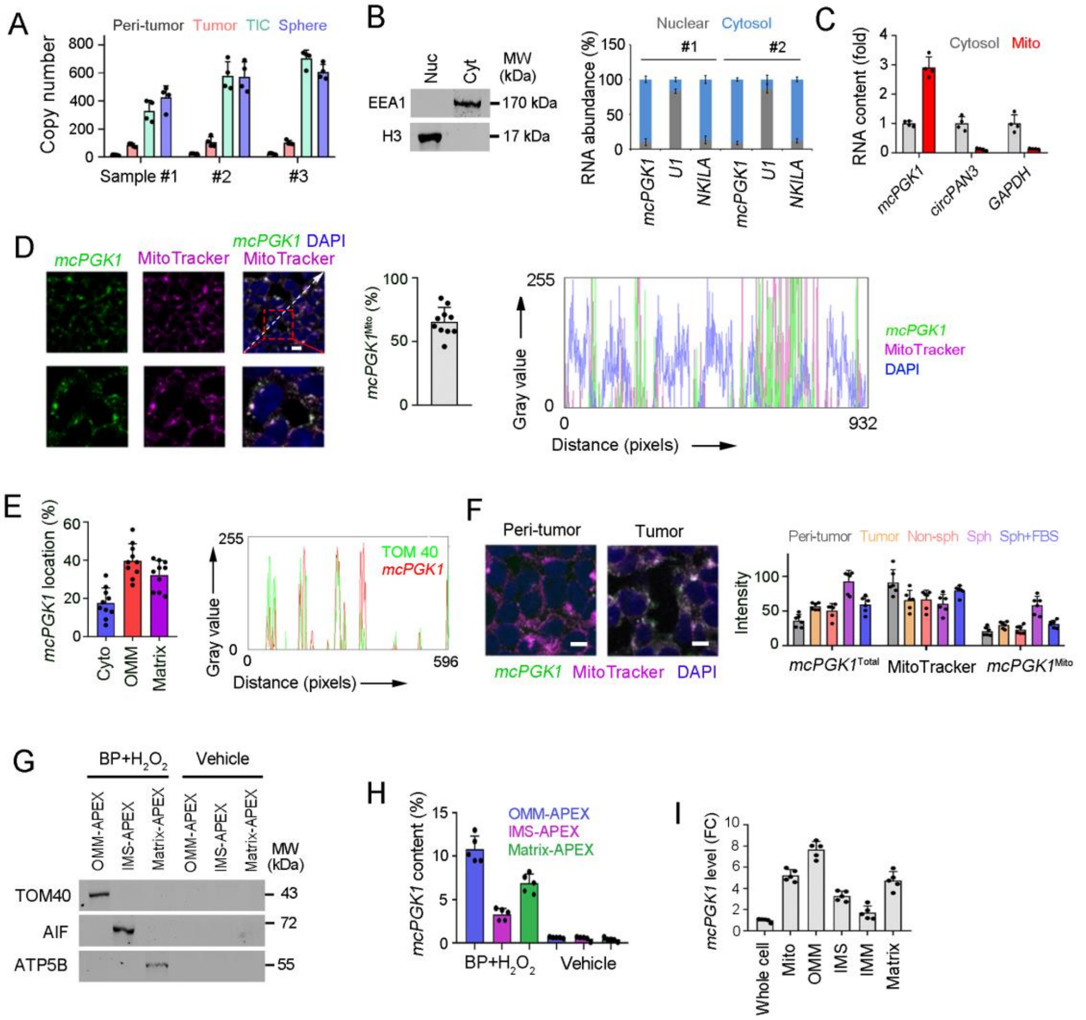

Supplementary Figure 3. Mitochondria-location of mcPGK1, related to Figure 1. (A) mcPGK1 copy number detection in peti-tumour cells, tumour cells, TICs and spheres. (B) Nucleocytoplasmic fractionation of primary cells was performed, followed by immunoblotting (left panel) and quantitative real-time PCR (right panel). U1 RNA served as a nuclear location control and NKILA was used as a cytoplasmic location control. The cytosol fractions contain mitochondria. (C) Quantitative real-time PCR for mcPGK1 in mitochondria fraction. Huh7 cells were used for mitochondria separation. *n*=4 independent experiments. (D) Confocal observation of mcPGK1 and mitochondria, showing the distribution of mcPGK1 in mitochondria. Typical images were shown in the upper panel, and mitochondrial mcPGK1 ratios and fluorescence signal analysis based on dashed white line were in the lower panel. *n*=10 images were observed for analysis. Scale bars, 10 μm. (E) mcPGK1 ratios and fluorescence signal analysis from *n*=10 structured illumination microscopy images. (F) Peri-tumour, tumour, non-sphere, sphere and sphere+FBS cells were used for mcPGK1 *in situ* hybridizsation and MitoTracker staining. Typical peri-tumour and tumour images were in left panels and statistical results were in right panels. Scale bars, 5 μm. (G) Western confirmation of APEX-based proteomic tagging. OMM, outer mitochondrial membrane; IMS, intermembrane space. TOM40, AIF, and ATP5B were used to represent OMM proteins, IMS, and mitochondrial matrix,

respectively. (H) mcPGK1 abundance in sub-mitochondrial fractions tagged by APEX labeling was detected by qRT-PCR. Oncosphere cells were used for APEX assay and *n*=5 independent experiments. BP, biotin-phenol. (I) Mitochondrial fractions were purified and mcPGK1 levels were detected via qRT-PCR. mcPGK1 levels were detected via qRT-PCR and normalized to whole cell levels. *n*=5 independent experiments. For all panels, data are shown as mean + s.d. Source data are provided as a Source Data file.

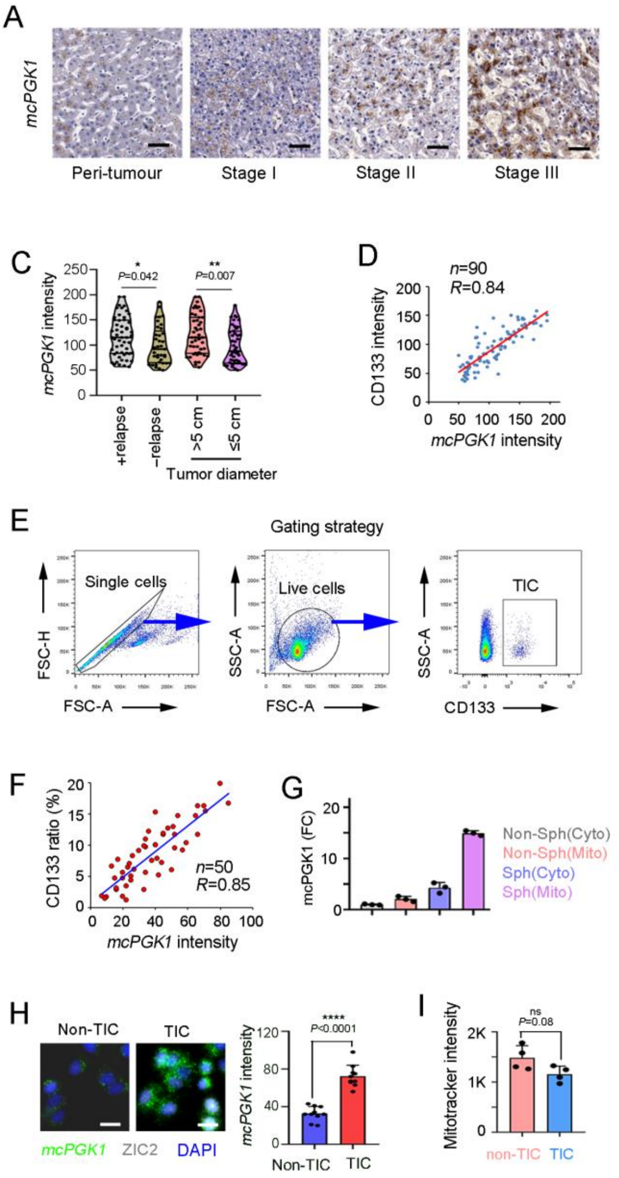

B

Variables	No. of patients	mcPGK1 intensity (mean ± SD)	P
Age (year)			
≥ 60	22	108.8 ± 32.5	0.91
< 60	68	109.9 ± 40.8	
Gender			
Female	16	105.9 ± 43.0	0.71
Male	74	110.4 ± 37.9	
Diameter			
≥ 5 (cm)	46	120.3 ± 39.2	0.007
< 5 (cm)	44	98.5 ± 35.3	
Tumor number			
≥ 1	16	118.6 ± 49.2	0.43
1	74	107.7 ± 36.0	
Cirrhotic tubercle			
> 3 (mm)	40	113.6 ± 39.6	0.39
≤ 3 (mm)	50	106.4 ± 38.1	
Stage			
I+II	65	93.4 ± 29.8	<0.001
III	25	151.6 ± 26.1	
Relapse			
Positive	53	116.5 ± 38.6	0.04
Negative	37	99.6 ± 37.1	
Liver cirrhosis			
Positive	78	110.3 ± 39.5	0.66
Negative	12	105.3 ± 34.3	
HBsAg			
Positive	77	110.8 ± 40.1	0.11
Negative	13	96.8 ± 27.3	
HBcAb			
Positive	79	111.0 ± 40.0	0.46
Negative	9	102.2 ± 29.5	
Total bilirubin (umol/L)			
≥ 14.67	43	114.1 ± 39.5	0.30
< 14.67	47	105.5 ± 37.9	
ALT (U/L)			
≥ 70.31	18	108.9 ± 39.9	0.94
< 70.31	72	109.8 ± 38.7	
ALB (g/dL)			
≥ 4.41	48	104.3 ± 40.7	0.17
< 4.41	42	115.6 ± 30.8	
AFP (ug/L)			
≥ 5395	14	116.2 ± 37.4	0.49
< 5395	76	108.4 ± 39.1	
GGT (U/L)			
≥ 87.5	29	110.8 ± 38.6	0.84
< 87.5	61	109.0 ± 39.0	

Supplementary Figure 4. mcPGK1 was highly expressed in liver cancer and liver TICs, related to Figure 1. (A) *In situ* hybridization of mcPGK1 in tissue microarray containing 90 liver tumours and 90 peri-tumour samples. Typical images of peri-tumours, stage I, stage II and stage III tumours were shown. Scale bars, 50 μm. (B) Clinical relation of mcPGK1 expression. All samples were divided into two groups according to the indicated clinical characters. (C) Violin plot of mcPGK1 expression in the indicated HCC groups. Medium, minimum, maximum and quarter levels were shown, as well as individual levels. (D) Co-expression of mcPGK1 and CD133. mcPGK1 and CD133 intensities were served as X-axis and Y-axis. (E) Gating strategy for CD133$^+$ TIC detection, related to Fig. 1D, Peti-tumour panel. (F) Positive correlation between *mcPGK1* intensity and CD133 ratios. *n*=50 HCC samples. (G) Quantitative real-time PCR detection for mcPGK1 expression in mitochondrial and cytosol fractions, which were isolated from non-sphere and sphere cells. *n*=3 independent experiments. (H) Fluorescence *in situ*

hybridization of mcPGK1 in TICs and non-TICs, with ZIC2 as a liver TIC marker. Typical images were shown in the left panel and statistical results of n=10 fields were shown in the right panel. Scale bars, 10 μm. (I) Mitochondrial levels in non-TICs and TICs were detected with MitoTracker FACS assay, and statistical results of MitoTracker intensity were shown. For all panels, data are shown as mean + s.d., *$P < 0.05$; **$P < 0.01$; ****$P < 0.0001$; ns, not significant, by two-tailed Student's T-test. Source data are provided as a Source Data file.

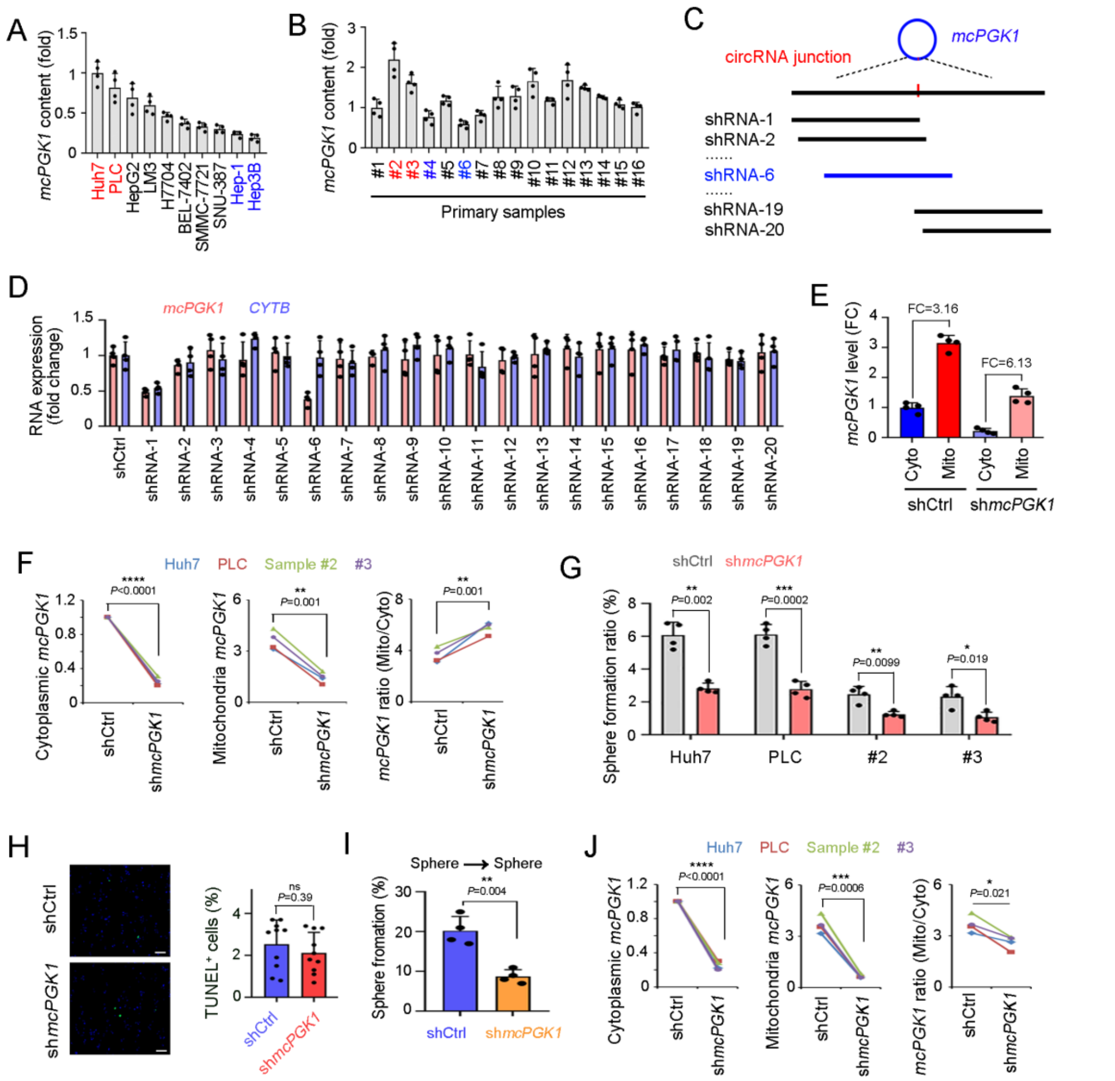

Supplementary Figure 5. mcPGK1 knockdown inhibits liver TIC self-renewal, related to Figure 2. (A, B) Expression levels of mcPGK1 in the indicated liver cell lines (A) and primary samples (B). High-expressing cells and low-expressing cells were labeled red and blue, respectively. (C, D) Identification of efficient shRNA for mcPGK1 knockdown. All shRNAs targeting mcPGK1 conjunction sequence were designed (C), and their involvement in mcPGK1 and CYTB expression was measured via real-time PCR (D). (E, F) Cytoplasm (Cyto) and mitochondria (Mito) fractions were isolated from mcPGK1 silenced and control cells, and mcPGK1 levels were analyzed via real-time PCR and normalized to those in Cytoplasm faction of shCtrl cells. Typical results were in E, and statistical data were in F. (G) Sphere formation ratios of mcPGK1 silenced and control cells. mcPGK1 was silenced in the indicated liver cancer cells via PSiCoR lentivirus. (H) TdT mediated dUTP Nick End Labeling (TUNEL) of mcPGK1 silenced and control Huh7 cells. Typical images were in the left panel and calculated ratios were in the right panel. Scale bars, 100 μm. (I) 1000 single cells isolated from Huh7 spheres were used for sphere formation assay. *n*=4 independent experiments. (J) Real-time PCR analyses for mcPGK1 levels in cytoplasm (Cyto) and mitochondria (Mito) fractions, which were from

mcPGK1 silenced control cells induced by Mito-NP. For A, B, D, E, G, *n*=4 independent experiments; for F, J, *n*=4 independent samples were examined; for H, *n*=10 fields per group. For all panels, data are shown as mean + s.d. **P* < 0.05; ***P* < 0.01; ***P* < 0.001; *****P* < 0.0001; ns, not significant, by two-tailed Student's T-test. Source data are provided as a Source Data file.

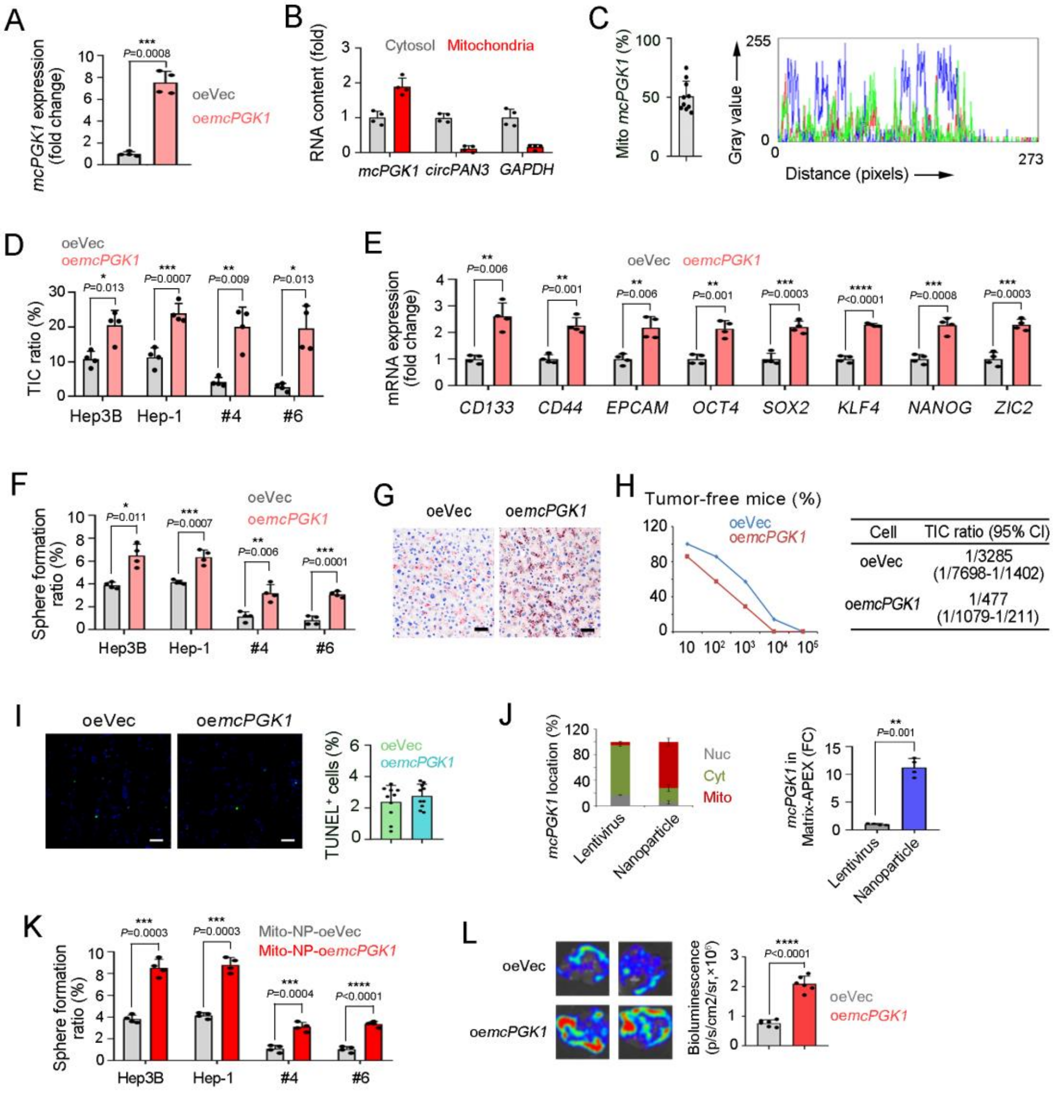

Supplementary Figure 6. mcPGK1 overexpression promotes liver TIC self-renewal, related to Figure 2. (A) Quantitative real-time PCR to detect the efficiency of mcPGK1 overexpression. oemcPGK1, mcPGK1 overexpression. *n*=4 independent experiments. (B, C) Quantitative real-time PCR (B) and confocal observation (C) to confirm the mitochondrial location of highly expressed mcPGK1. For B, *n*=4 independent experiments. For C, *n*=10 fields. (D) $CD133^+$ TIC ratios in mcPGK1 overexpressing (oemcPGK1) and control cells. *n*=4 independent experiments. (E) Quantitative real-time PCR for the expression levels of the indicated TIC marker genes and TIC-related TFs. *n*=4 independent experiments. (F) Sphere numbers of mcPGK1 overexpressing and control cells. *n*=30 spheres from four independent experiments were measured. (G, H) Tumour initiation assay of gradient numbers of mcPGK1 overexpressing cells. *n*=7 6-week-old male BALB/c nude mice per group. For G, mcPGK1 overexpression efficiency was examined by RNAscope. Scale bars, 30 μm. For H, the ratios of tumour-free mice were in left panel, TIC ratios and *P* value calculated by ELDA were in right panel. (I) TUNEL of mcPGK1 overexpressing cells (oemcPGK1) and control (oeVec) cells. Typical images and

TUNEL$^+$ cell ratios of *n*=10 images were shown. Scale bars, 100 μm. (J) The subcellular location of mcPGK1 was detected via quantitative real-time PCR (left) or Matrix-APEX assay. *n*=4 independent experiments. (K) Sphere formation assay using mcPGK1 overexpressing and control cells. *n*=4 independent experiments were performed with similar results. (L) 1×10^6 mcPGK1 overexpressing and control luciferase-labeled primary #2 cells were used for in vivo propagation, and typical liver images and calculated results were shown. *n*=6 mice were used per group. In all panels, data are shown as mean + s.d. *P < 0.05; **P < 0.01; ***P < 0.001; ****P < 0.0001, by two-tailed Student's T-test. Source data are provided as a Source Data file.

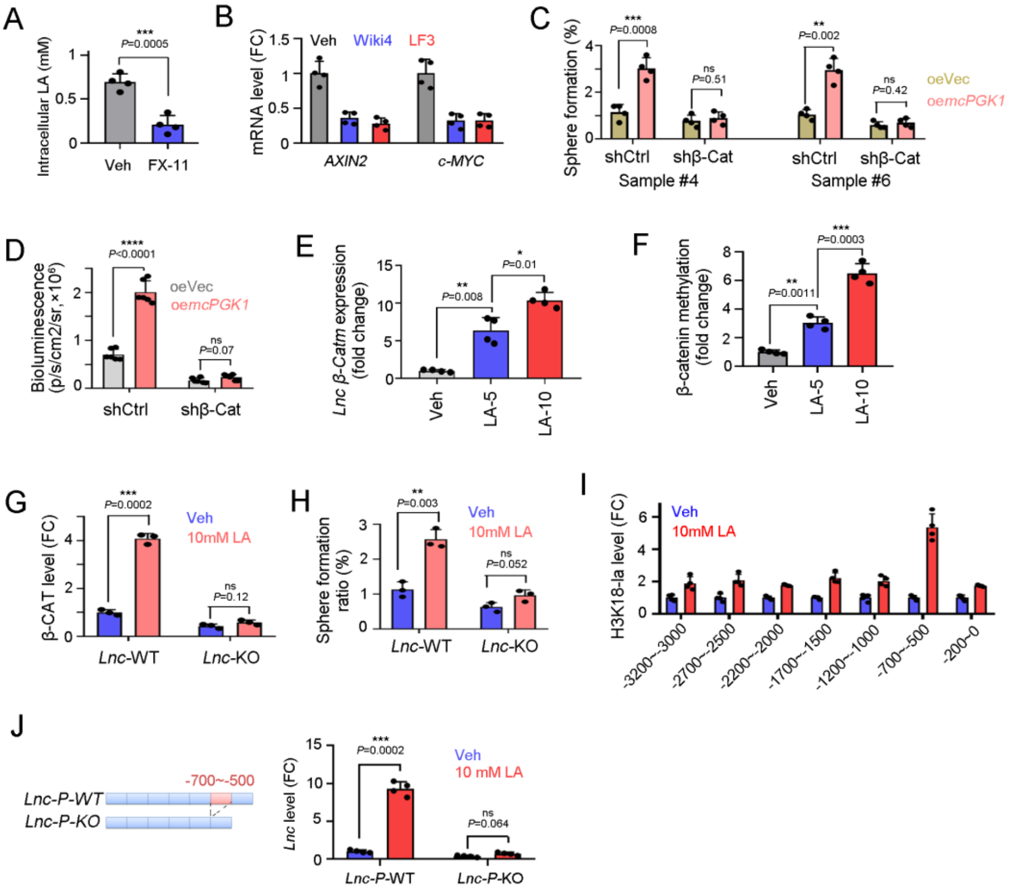

Supplementary Figure 7. Molecular mechanisms of lactic acid in β-catenin regulation, related to Figure 4, 5. (A) Primary sample #2 cells were treated with 20 μmol/L FX-11 and intracellular LA concentration was detected. (B) Primary sample #2 cells were treated with 100 nM Wiki4 and 10 μm LF3 for 2 days, and the activation of Wnt/β-catenin signaling pathway was detected by quantitative real-time PCR analysis of *AXIN2* and *c-MYC*. (C) mcPGK1 was overexpressed in β-catenin silenced and control cells, followed by sphere formation assay. (D) 5×10^6 luciferase reporter cells were used for in vivo propagation. *n*=6 mice were used per group. (E) Expression levels of *lnc-β-Catm* in the indicated cells were examined by real-time PCR. (F) Lactic acid treated primary sample #2 cells were used for β-catenin methylation detection through immunoprecipitation and Western blot. (G) *lnc-β-catm* knockout (Lnc-KO) and control WT (Lnc-WT) cells were treated with 10 mM lactic acid for 3 days, followed by β-catenin detection. (H) 5000 Lnc-KO and Lnc-WT cells were used for sphere formation assay, supplemented with or without 10 mM lactic acid. (I) Primary sample #2 cells were treated with 10 mM lactic acid for 3 days, followed by H3K18-La chromatin immunoprecipitation (ChIP). (J) The -700~-500 fragment of *lnc-β-catm* promoter was deleted (*Lnc-P*-KO) and treated with 10 mM lactic acid for 3 days, and then *lnc-β-catm* expression was examined through real-time PCR assay. For A, B, C, E, F, I, J, *n*=4 independent experiments; for G, H, *n*=3 independent experiments. In all panels, data are shown as mean + s.d. **P* < 0.05; ***P* < 0.01; ****P* < 0.001; *****P* < 0.0001; ns, not significant, by two-tailed Student's T-test. Source data are provided as a Source Data file.

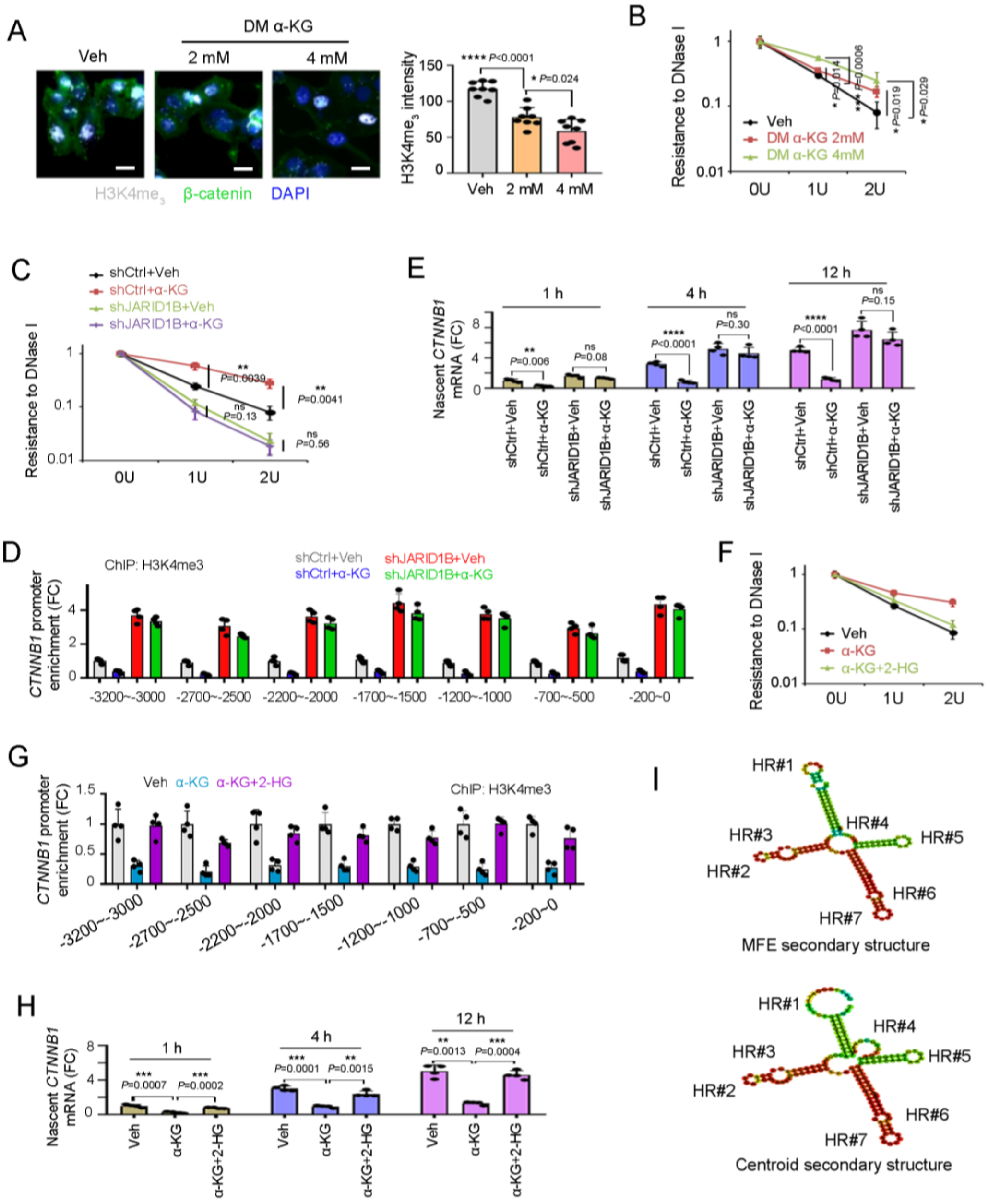

Supplementary Figure 8. The role of mcPGK1-PDK1-PDH in metabolic reprogramming, related to Figure 5, 6. (A) H3K4me3 levels in indicated cells were analyzed via immunofluorescence. *n*=8 fields per group. Scale bars, 10 μm. (B) DNase I resistance of *CTNNB1* promoter in indicated cells was analyzed through real-time PCR. (C) JARID1B silenced cells were generated and treated with 4 mM α-KG for 3 days, and the chromatin accessibility of *CTNNB1* promoter was measured by DNase sensibility. (D, E) JARID1B knockdown and control primary sample #2 cells were treated with 4 mM α-KG for 3 days. H3K4me3 levels at the indicated fragments of *CTNNB1* promoter were detected through ChIP assay (D), and nascent *CTNNB1* mRNA was measured through Click-it EU labeling assay (E). (F-H) Primary sample #2 cells were treated with 4 mM α-KG, or 4 mM α-KG plus 200 mM 2-HG for 3 days, the chromatin accessibility of -200~0 region of *CTNNB1* promoter was measured through DNase I treatment (F), H3K4me3

levels at *CTNNB1* promoter were measured using H3K4me3 ChIP assay (G), and nascent *CTNNB1* mRNA was measured via Click-it EU labeling assay (H). (I) Stem-loop structures of mcPGK1. Predictions were based on minimum free energy (MFE) and partition function (http://rna.tbi.univie.ac.at/). For all panels, data are shown as mean + s.d. For B-H, n=4 independent experiments. *P < 0.05; **P < 0.01; ***P < 0.001; ****P < 0.0001; ns, not significant, by two-tailed Student's T-test. Source data are provided as a Source Data file.

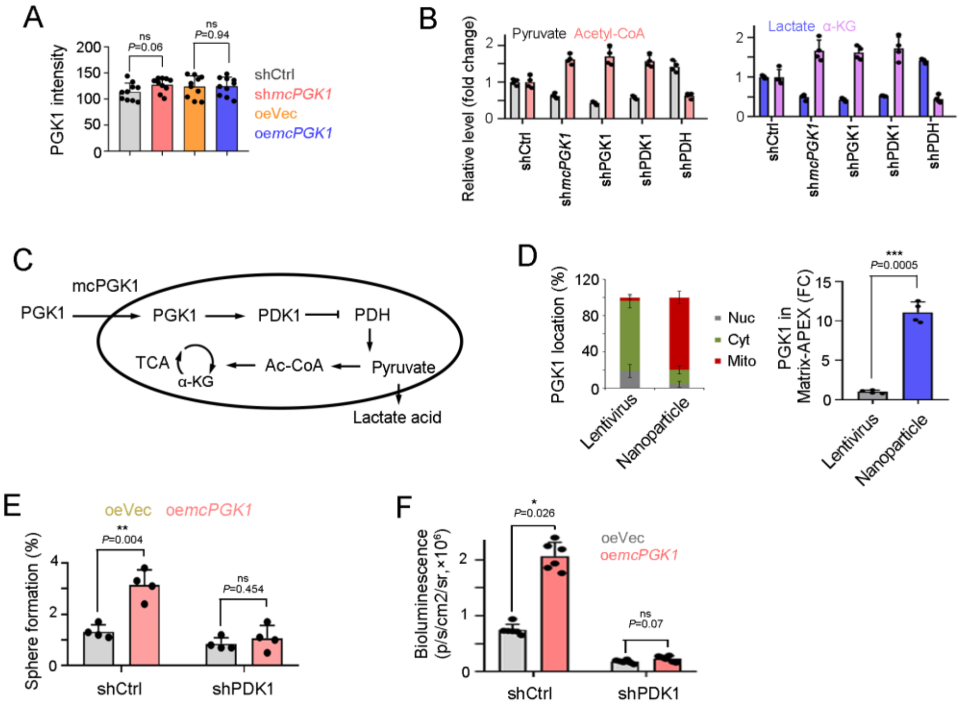

Supplementary Figure 9. *McPGK1* promotes the mitochondrial translocation of PGK1, related to Figure 8. (A) PGK1 intensity in the indicated cells. *n*=10 fields were used for PGK1 detection. (B) Liver cancer cells expressing shmcPGK1, shPGK1, shPDK1 or shPDH1 were cultured for 6 hr, and the levels of pyruvate, mitochondrial acetyl-CoA were measured (left panel), lactate and α-ketoglutarate (α-KG) (right panel) were measured. All levels were normalized to shCtrl cells. (C) Schematic diagram showing mcPGK1-PGK1-PDK1-PDH axis in metabolic reprogramming. (D) The subcellular location of PGK1 was detected via real-time PCR (left) or Matrix-APEX assay (right). Nuclear (Nuc), cytosol (without mitochondria) and mitochondrial fractions were isolated from lentvirus or nanoparticle treated cells, followed by real-time PCR detection (left). Mitochondrial factions were enriched through APEX and PGK1 levels were detected via real-time PCR (right). (E, F) PDK1 silenced #4 cells were generated and used for mcPGK1 overexpression, followed by sphere formation (E) and *in vivo* propagation assay (F). For all panels, data are shown as mean + s.d. For B, D, E, *n*=4 independent experiments; for F, *n*=6 mice per group. **P* < 0.05; ***P* < 0.01; ****P* < 0.001; ns, not significant, by two-tailed Student's T-test.. Source data are provided as a Source Data file.

Supplementary Table 1. shRNA sequence used in this study

Primers	Sequences
PGK1	5'- GGATGTCTATGTCAATGATGC-3'
PDK1	5'- CCGAACTAGAACTTGAAGA-3'
PDH	5'- GCAATCAGCTGCCTGCAAATG -3'

Supplementary Table 2. PCR primers used in this study

Primers	Sequences
18S (Forward)	5'-AACCCGTTGAACCCCATT-3'
18S (Reverse)	5'-CCATCCAATCGGTAGTAGCG-3'
actin (Forward)	5'-GGCTGTATTCCCCTCCATCG-3'
actin (Reverse)	5'-CCAGTTGGTAACAATGCCATGT-3'
CD133 (Forward)	5'-AGTCGGAAACTGGCAGATAGC -3'
CD133 (Reverse)	5'-GGTAGTGTTGTACTGGGCCAAT -3'
CD44 (Forward)	5'-CTGCCGCTTTGCAGGTGTA -3'
CD44 (Reverse)	5'-CATTGTGGGCAAGGTGCTATT -3'
EPCAM (Forward)	5'-AATCGTCAATGCCAGTGTACTT -3'
EPCAM (Reverse)	5'-TCTCATCGCAGTCAGGATCATAA -3'
OCT4 (Forward)	5'-CTGGGTTGATCCTCGGACCT-3'
OCT4 (Reverse)	5'-CCATCGGAGTTGCTCTCCA-3'
SOX2 (Forward)	5'-GCCGAGTGGAAACTTTTGTCG-3'
SOX2 (Reverse)	5'-GGCAGCGTGTACTTATCCTTCT-3'
KLF4 (Forward)	5'-CCCACATGAAGCGACTTCCC-3'
KLF4 (Reverse)	5'-CAGGTCCAGGAGATCGTTGAA-3'
NANOG (Forward)	5'-TTTGTGGGCCTGAAGAAAACT-3'
NANOG (Reverse)	5'-AGGGCTGTCCTGAATAAGCAG-3'
ZIC2 (Forward)	5'-GCGCAACTCCACAACCAGTA-3'
ZIC2 (Reverse)	5'-TGCCGCATATAGCGGAAAAAG-3'
