# Supplementary material for: mcPGK1-dependent mitochondrial import of PGK1 promotes metabolic reprogramming and self-renewal of liver TICs
Source: Nat Commun. 2023 Feb 27;14:1121. doi: 10.1038/s41467-023-36651-5 (PMC9971191; doi:10.1038/s41467-023-36651-5)
Supplement: Supplementary file 2 — Reporting Summary [file 41467_2023_36651_MOESM2_ESM.pdf]

nature portfolio

Corresponding author(s): Zhenzhen Chen, Benyu Liu, Pingping Zhu

Last updated by author(s): Jan 23, 2023

Reporting Summary

Nature Portfolio wishes to improve the reproducibility of the work that we publish. This form provides structure for consistency and transparency in reporting. For further information on Nature Portfolio policies, see our Editorial Policies and the Editorial Policy Checklist.

Statistics

For all statistical analyses, confirm that the following items are present in the figure legend, table legend, main text, or Methods section.

n/a	Confirmed	
☐	☒	The exact sample size (*n*) for each experimental group/condition, given as a discrete number and unit of measurement
☐	☒	A statement on whether measurements were taken from distinct samples or whether the same sample was measured repeatedly
☐	☒	The statistical test(s) used AND whether they are one- or two-sided<br>*Only common tests should be described solely by name; describe more complex techniques in the Methods section.*
☐	☒	A description of all covariates tested
☒	☐	A description of any assumptions or corrections, such as tests of normality and adjustment for multiple comparisons
☐	☒	A full description of the statistical parameters including central tendency (e.g. means) or other basic estimates (e.g. regression coefficient) AND variation (e.g. standard deviation) or associated estimates of uncertainty (e.g. confidence intervals)
☐	☒	For null hypothesis testing, the test statistic (e.g. *F*, *t*, *r*) with confidence intervals, effect sizes, degrees of freedom and *P* value noted<br>*Give P values as exact values whenever suitable.*
☒	☐	For Bayesian analysis, information on the choice of priors and Markov chain Monte Carlo settings
☐	☒	For hierarchical and complex designs, identification of the appropriate level for tests and full reporting of outcomes
☒	☐	Estimates of effect sizes (e.g. Cohen's *d*, Pearson's *r*), indicating how they were calculated

Our web collection on statistics for biologists contains articles on many of the points above.

Software and code

Policy information about availability of computer code

Data collection	No software was used for data collection.
Data analysis	Data were analyzed by GraphPad Prism 5.0, SPSS 20.0 and Microsoft Excel 2010. All flow cytometry data were analyzed with FlowJo 10 (Treestar). Adobe Photoshop CC 14.0, ImageJ 1.48, Microsoft PowerPoint 2010 and Image-Pro Plus 6.0 were used for image analysis and Figure presentation. Online-available tool ELDA (https://bioinf.wehi.edu.au/software/elda/) was used for TIC ratios. Two-tailed unpaired Student's T-test was performed using Microsoft Excel 2010. No customized computer code was used.

For manuscripts utilizing custom algorithms or software that are central to the research but not yet described in published literature, software must be made available to editors and reviewers. We strongly encourage code deposition in a community repository (e.g. GitHub). See the Nature Portfolio guidelines for submitting code & software for further information.

Data

Policy information about availability of data

All manuscripts must include a data availability statement. This statement should provide the following information, where applicable:

- Accession codes, unique identifiers, or web links for publicly available datasets
- A description of any restrictions on data availability
- For clinical datasets or third party data, please ensure that the statement adheres to our policy

The circRNA sequencing data generated in this study have been deposited in the Gene Expression Omnibus (GEO) Database under accession code GSE223661 [https://www.ncbi.nlm.nih.gov/geo/query/acc.cgi?acc=GSE223661]. Source data generated in this study are provided in the Source Data file.

Human research participants

Policy information about studies involving human research participants and Sex and Gender in Research.

Reporting on sex and gender	Liver tumour tissues were randomly collected without bias. There are 90 liver tumour samples in tissue microarray, including 16 female and 74 male samples.
Population characteristics	There is no population characteristics analysis in the present manuscript.
Recruitment	There is no potential self-selection bias or other biases that could affect the results.
Ethics oversight	Ethics Committee of Zhengzhou University.

Note that full information on the approval of the study protocol must also be provided in the manuscript.

Field-specific reporting

Please select the one below that is the best fit for your research. If you are not sure, read the appropriate sections before making your selection.

☒ Life sciences ☐ Behavioural & social sciences ☐ Ecological, evolutionary & environmental sciences

For a reference copy of the document with all sections, see nature.com/documents/nr-reporting-summary-flat.pdf

Life sciences study design

All studies must disclose on these points even when the disclosure is negative.

Sample size	For Figure 1D, n=50 samples were detected. For Figure 1G, 1H, 2F, 5D, 8A, 8B, S2G, S3D, S3E, S4H, S5H, S6C, S6I, S9A, n=10 fields were taken for statistical analysis. For Figure 2D, 4B, n=30 spheres from 3 independent experiments were measured. For Figure 2H, S1C and S6H, n=7 BALB/c nude mice were used per group. For Figure 4G and 6H, n=6 BALB/c nude mice were used per group. For Figures S4A, S4B, S4C, tissue microarray containing n=90 pairs of tumour and peri-tumours was used. For other figures, we performed at least three independent experiments . All experiments were conducted with multiple biological replicates. Sample sizes were chosen based on previous experience with specific experimental setup (Zhu P et al, Neuron 2022; Zhu P et al, Nat Struc Mol Biol 2016; Chen Z et al, J Clin Invest 2021; Chen Z et al, Cell Rep 2021); no statistical method was used to determine sample size. All the sample size is acceptable for the field. We showed the values of individual samples in Figures and also stated the extract sample sizes in Figure legends.
Data exclusions	For mice experiments, we used littermates with the same age and gender for each group, and excluded the mice 5 g thinner than other littermates before any treatment or analysis. For human cells, no data have been excluded.
Replication	Data are representative of at least three independent experiments, and at least three mice or cells were used, and all the results are repeatable. For further verification, we repeated the key results (eg, the role of mcPGK1 in the mitochondrial entry of PGK1, metabolic reprogramming and TIC self-renewal) by independent individuals in our lab and cooperative labs. All the results are reproducible, and the Figures are representative results.
Randomization	All mice and samples were distributed into treatment groups. In different groups, the average weight of mice are similar and the numbers are the same. All samples were selected randomly and observed without prejudice. All images shown in Figures are representative images. We also stated this point in Figure legends.
Blinding	All experiments are not performed in blind at the beginning, because the investigator who participated in experimental design also performed the experiment. However, we repeated the key findings in blind, including the role of mcPGK1 in the mitochondrial entry of PGK1, metabolic reprogramming and TIC self-renewal. The investigators were blinded to the kind of cells during these experiments. For other experiments, the investigator was non-blinded, and we observed and analyzed the results according to standard pipeline, without prejudice.

Reporting for specific materials, systems and methods

We require information from authors about some types of materials, experimental systems and methods used in many studies. Here, indicate whether each material, system or method listed is relevant to your study. If you are not sure if a list item applies to your research, read the appropriate section before selecting a response.

Materials & experimental systems

n/a	Involved in the study
☐	☒ Antibodies
☐	☒ Eukaryotic cell lines
☒	☐ Palaeontology and archaeology
☐	☒ Animals and other organisms
☒	☐ Clinical data
☒	☐ Dual use research of concern

Methods

n/a	Involved in the study
☒	☐ ChIP-seq
☐	☒ Flow cytometry
☒	☐ MRI-based neuroimaging

Antibodies

Antibodies used

Anti-β-Catenin (catalog no. 610153) and anti-CD133 antibody (catalog no. 566598) was purchased from BD Bioscience. Anti-PGK1 (catalog no. 68540S), anti-EEA1 (catalog no. 3288S), anti-β-actin (catalog no. 4970), anti-H3 (catalog no. 4499) and anti-H3K4me3 (catalog no. 9751S) antibodies were from Cell Signaling Technology. Anti-ZIC2 (catalog no. ARP35821_P050) antibody was purchased from Aviva Systems Biology. Anti-TOM40 (catalog no. 18409-1-AP), anti-TOM70 (catalog no. 14528-1-AP), anti-c-MYC (catalog no. 10828-1-AP) and anti-AXIN2 (catalog no. 20540-1-AP) antibodies were from Proteintech Group, Inc. Goat anti-Mouse IgG (H+L) Cross-Adsorbed Secondary Antibody, Alexa Fluor™ 594 antibody (catalog no. A-11005), Goat anti-Rabbit IgG (H+L) Cross-Adsorbed Secondary Antibody, Alexa Fluor™ 488 (catalog no. A-11008), Goat anti-Rabbit IgG (H+L) Cross-Adsorbed Secondary Antibody, Alexa Fluor™ 647 (catalog no. A-21244) were purchased from Invitrogen. HRP-conjugated Affinipure Goat Anti-Mouse IgG(H+L) antibody (catalog no. SA00001-2) and HRP-conjugated Affinipure Goat Anti-Rabbit IgG(H+L) antibody (catalog no. SA00001-2) were purchased from Proteintech Group, Inc.

Validation

All antibodies were verified by manufacturers and widely used in the published literatures. A full reference list can be found on the official website of the manufacturer. We also examined antibodies according to the manuals in preliminary experiments, and selected the antibodies those got similar results with validation results on manufacturers' websites or relevant citations. Primary antibody links containing species, applications, dilutions, validations and citations are provided below for each antibody:
Anti-β-Catenin (catalog no. 610153): https://www.bdbiosciences.com/zh-cn/search-results?searchKey=610153
Anti-CD133 antibody (catalog no. 566598): https://www.bdbiosciences.com/zh-cn/search-results?searchKey=566598
Anti-PGK1 (catalog no. 68540S): https://www.cellsignal.cn/products/primary-antibodies/pgk1-antibody/68540
Anti-EEA1 (catalog no. 3288S): https://www.cellsignal.cn/products/primary-antibodies/eea1-c45b10-rabbit-mab/3288
Anti-β-actin (catalog no. 4970): https://www.cellsignal.cn/products/primary-antibodies/b-actin-13e5-rabbit-mab/4970
Anti-H3 (catalog no. 4499): https://www.cellsignal.cn/products/primary-antibodies/histone-h3-d1h2-xp-rabbit-mab/4499
Anti-H3K4me3 (catalog no. 9751S): https://www.cellsignal.cn/products/primary-antibodies/tri-methyl-histone-h3-lys4-c42d8-rabbit-mab/9751
Anti-ZIC2 (catalog no. ARP35821_P050): https://www.avivasysbio.com/zic2-antibody-c-terminal-region-arp35821-p050.html
Anti-TOM40 (catalog no. 18409-1-AP): https://www.ptgcn.com/products/TOMM40-Antibody-18409-1-AP.htm
Anti-TOM70 (catalog no. 14528-1-AP): https://www.ptgcn.com/products/TOM70-Antibody-14528-1-AP.htm
anti-c-MYC (catalog no. 10828-1-AP): https://www.ptgcn.com/products/MYC-Antibody-10828-1-AP.htm
Anti-AXIN2 (catalog no. 20540-1-AP): https://www.ptgcn.com/products/AXIN2-Antibody-20540-1-AP.htm

Eukaryotic cell lines

Policy information about cell lines and Sex and Gender in Research

Cell line source(s)

Human cell lines Hep3B were obtained from ATCC (catalog no, HB-8064), Huh7 cells were obtained from iCellbioscience (catalog no. iCell-h080), 293T, PLC and Hep-1 cells were from Zusen Fan lab (Institute of Biophysics, Chinese Academyof Sciences).

Authentication

All cells were authenticated through realtime-PCR with the respective marker genes, and the cells are consistent with morphological and functional descriptions.

Mycoplasma contamination

We have tested these cell lines and found no mycoplasma contamination.

Commonly misidentified lines
(See ICLAC register)

No commonly misidentified lines were used in functional detection.

March 2021

Animals and other research organisms

Policy information about studies involving animals; ARRIVE guidelines recommended for reporting animal research, and Sex and Gender in Research

Laboratory animals	Unless specifically stated, all the mice we used were 6-week male BALB/c nude mice. Mice were housed in groups of 4-7 mice per cage in 12 h light/dark cycle (7:00-19:00 light, 19:00-7:00 dark), with controlled room temperature (23±2℃) and humidity (40-60%). We complied the ethical regulations of the ethical committee of Zhengzhou University, and complied the ARRIVE guidelines. The study is compliant with all relevant ethical regulations regarding animal research.
Wild animals	No wild animals were used in this study.
Reporting on sex	Male BALB/c nude mice were used for liver tumour initiation assay. We also stated it in the Methods and Figure legends.
Field-collected samples	This study did not involve sample collected from the field.
Ethics oversight	All SPF mice were bred and housed in SPF condition in School of Life Sciences, Zhengzhou University, and were used according to protocols approved by the ethical committee of Zhengzhou University (ZZUIRB202054 and ZZUIRB202055).

Note that full information on the approval of the study protocol must also be provided in the manuscript.

Flow Cytometry

Plots

Confirm that:

☒ The axis labels state the marker and fluorochrome used (e.g. CD4-FITC).

☒ The axis scales are clearly visible. Include numbers along axes only for bottom left plot of group (a 'group' is an analysis of identical markers).

☒ All plots are contour plots with outliers or pseudocolor plots.

☒ A numerical value for number of cells or percentage (with statistics) is provided.

Methodology

Sample preparation	Fresh liver cancer tissues were washed two or three times and kept in DMEM/F12 medium supplemented with 1000 U/ml penicillin and 1000 U/ml streptomycin, and transferred on ice. Samples were then washed with pre-cooled sterile PBS supplemented with 100 U/ml penicillin and 100 U/ml streptomycin, cut into small fragments, and digested in HBSS containing 0.03% pronase, 0.05% type IV collagenase, and 0.01% deoxyribonuclease for 30 min at 37 °C. Then sample was filtered through 100 μm nylon filter, centrifuged for 2 min at 50 x g in 4 °C and HCC primary cells were in precipitation. Then liver cancer cells were stained with BV421 conjugated anti-CD133 antibody, and CD133+ liver TICs were analyzed or enriched by FACS.
Instrument	BD FACSCalibur was used for detection and BD FACSAria IIIu for sorting.
Software	Flow cytometry data were analyzed with FlowJo 10 (Treestar).
Cell population abundance	CD133+ TICs were grouped separately from CD133- non-TIC cells, and can be detected/analyzed directly. IgG control antibody was also served as negative control. About 1%~20% cells were CD133+ TICs, and the ratios vary greatly in different samples. Huh7, 10-15%; PLC, about 20%; Hep3B, about 10%; Hep-1, about 10%; #2, about 10%; #3, about 10%; #4, about 3%; #6, about 2%.
Gating strategy	Gating strategy was shown in supplementary Fig. 4E.

☒ Tick this box to confirm that a figure exemplifying the gating strategy is provided in the Supplementary Information.
